# The molecular circadian clock of eosinophils: A potential therapeutic target for asthma

**DOI:** 10.1152/ajpcell.00149.2025

**Published:** 2025-03-25

**Authors:** Julia Teppan, Thomas Bärnthaler, Aitak Farzi, Hannah Durrington, Gael Gioan-Tavernier, Hazel Platt, Peter Wolf, Akos Heinemann, Eva Böhm

**Affiliations:** 1 Otto Loewi Research Center for Vascular Biology, Immunology and Inflammation, Division of Pharmacology, Medical University of Graz, 8010 Graz, Austria; 2 Lung Research Cluster, Medical University of Graz, 8010 Graz, Austria; 3 Department of Dermatology and Venerology, Medical University of Graz, 8010 Graz, Austria; 4 Faculty of Biology, Medicine and Health, University of Manchester, Manchester, UK; 5 Manchester University NHS Foundation Trust, Manchester, UK

**Keywords:** asthma, eosinophils, molecular circadian clock, retinoic acid receptor-related orphan receptor, asthma treatment

## Abstract

Asthma is a chronic inflammatory airway disease exhibiting time-of-day variability in symptoms and severity. Eosinophils, pivotal players and biomarkers in asthma, are regulated by the molecular circadian clock. This study aimed to investigate the impact of the molecular circadian clock on eosinophil effector function and its potential as a diagnostic biomarker and therapeutic target.

We monitored clock proteins by flow cytometry in peripheral blood eosinophils from mild asthmatics over a 24-hour period. The observed decreased protein levels were confirmed in a cohort of patients with moderate asthma. To assess the interaction between inflammation and the molecular circadian clock, eosinophils were stimulated with patients’ sera, inflammatory mediators, and clock-modulating ligands. The therapeutic potential of the inverse ROR agonist SR1001 was evaluated *in vitro* and in a murine model of allergen-induced airway inflammation.

Altered protein levels of CLOCK, BMAL1, REV-ERBs, and RORs in eosinophils from asthmatics reflected the disease severity and allergy status of the patients. Mimicking an inflammatory environment in vitro resulted in similar changes. Blocking CCR3/ERK and EGFR signaling with an inverse ROR agonist SR1001 reset the molecular circadian clock in eosinophils and exhibited anti-inflammatory effects by inhibiting eosinophil migration *in vitro*. Additionally, we confirmed the therapeutic potential of the clock-modulating SR1001, bronchoprotective effects in two *in vivo* models. This study suggests that clock proteins could serve as therapeutic targets in asthma. Pharmacological inhibition of ROR signaling demonstrated significant anti-inflammatory and bronchoprotective properties, indicating its potential as a novel treatment strategy for asthma and other eosinophilic diseases.

## 1 Introduction

Asthma is an inflammatory airways disease with a clear circadian signature. Airway inflammation and constriction follow a diurnal pattern and symptoms often worsen at night. Diagnostic markers such as forced expiratory volume in 1 second (FEV1) and fractional exhaled nitric oxide (FeNO) also reflect this circadian variation. FEV1 reaches its nadir in the early morning, while a rhythmic cycle for FeNO is only seen in asthmatics, with a peak at 10 a.m. and nadir overnight (1–5). Additionally, a nocturnal peak in eosinophils, neutrophils, and lymphocytes is observed in sputum of asthmatics, along with elevated serum concentrations of IL-5 and eotaxin in the early morning (3, 6–9).

Thus, the migration of leukocytes into the lung is timed by a network of interacting transcriptional/translational feedback loops, the molecular circadian clock (MCC) (9, 10). These loops are connected by the core transcription factors BMAL1 and CLOCK, which, as a heterodimer, serve as the main orchestrators of the MCC. The so-called accessory loop comprises two competing nuclear receptor families: nuclear receptor subfamily 1 group D (NR1D) also known as REV ERB (abbreviation for “reverse strand of the thyroid hormone receptor-α gene ERBA”) and the retinoic acid receptor-related orphan receptors (ROR). While ROR directly activates the transcription of the *BMAL1* gene, REV ERB functions as its repressor and as an intermediary between the immune system and the MCC (11, 12). Baumann and colleagues were the first to detect the MCC at the RNA level in human eosinophils (13).

Various immune functions, such as leukocyte trafficking and cytokine release, show daily variation, which is crucial for an effective immune response (9, 10, 14). Thus, disturbances within the circadian system can induce or progress inflammatory diseases (15, 16). Conversely, under inflammatory conditions, pro-inflammatory cytokines can affect the circadian clock and clock-controlled processes such as metabolism and sleep-wake cycle (17, 18). Furthermore, night shift workers are more likely to develop moderate to severe asthma (19) and altered clock gene expression has been found in airway brushings and peripheral leukocytes from asthmatic patients (16, 20).

Despite recent advances in asthma therapy and an increasing range of therapeutics there are still patients with difficult-to-treat or uncontrolled asthma (21). Therefore, we explored the function of the MCC in blood eosinophils from asthmatic patients, evaluated its association with disease severity, and assessed the therapeutic effects of clock-modulating synthetic ligands. Our findings show that the MCC responds to inflammatory stimuli, is damped in eosinophils from asthmatics, and reflects asthma severity. Resetting the MCC with an inverse ROR agonist inhibited crucial signaling pathways, prevented airway inflammation, and improved lung function parameters.

## 2 Material and Methods

### 2.1 Ethical Approval

Experiments with human material were authorized by the Institutional Review Boards and performed with written informed consent of all donors. Blood donors were assigned based on a clinical diagnosis following the Global Initiative for Asthma (GINA) guidelines (22), total and specific Immunoglobulin E (IgE), and reported symptoms. Animal experiments were approved by the Austrian Federal Ministry of Science and Research's Animal Ethics Committee. Further information is provided in the Supplemental material.

### 2.2 Monitoring experiment

We conducted a 24-hour flow cytometric monitoring experiment to validate clock protein oscillations in peripheral blood leukocytes, collecting blood at 4 a.m., 12 p.m., and 8 p.m. Samples were analyzed immediately or after 4 hours of incubation at 37°C, resulting in six time points of evaluation (Figure S1). Preliminary experiments and previous studies confirm that the MCC remains active *in vitro/ex vivo* for several hours (15, 23).

### 2.3 Flow cytometric staining

Whole blood was stained for leukocyte populations using the following antibodies: CD3-APC-Cy7, CD14-BV421 and CD16-PerCP-Cy5.5 (all from Biolegend). Cells were treated with FIX&PERM® (Mubio), blocked with FC-block, stained with primary antibodies against BMAL1 (Novusbio), CLOCK (Mybiosource), REV ERBα (Abcam), REV ERBβ (Novusbio), RORα (Thermo Fisher), RORβ (Novusbio), and RORγ (R&D Systems) followed by a PE-labeled secondary antibody (Biolegend) (15).

### 2.4 Isolation of human peripheral blood polymorphonuclear leukocyte (PMNL) and eosinophils

PMNLs were isolated via dextran sedimentation and density gradient centrifugation. Eosinophils were purified using the Human Eosinophil Isolation Kit (Miltenyi Biotec) following the manufacturer's protocol (24).

### 2.5 Cell isolation from sputum

Cell plugs were separated from saliva and incubated with 1:10 diluted DTT in a 1:4 ratio. After adding another four volumes of PBS, samples were filtered through a pre-wet mesh and centrifuged.

### 2.6 Immunofluorescence microscopy

Lung biopsy samples were deparaffinized and antigen-retrieved. After blocking unspecific binding, sections were incubated overnight with primary antibodies against BMAL1 and EPX (5 μg/ml). Secondary antibodies were applied, and nuclear staining was done with DAPI. Images were captured using consistent laser settings on a confocal microscope. Control slides were stained with secondary antibodies only.

### 2.7 Cytokine Multiplex

Cytokines in serum were determined by the HU Th Cytokine Panel (12-plex, Biolegend) according to the manufacturer’s instructions.

### 2.8 Pre-treatments

Cells were stimulated for 3 hours at 37°C with serum from asthma patients and pro-/anti-inflammatory mediators, and pretreated with clock-modulating ligands targeting REV ERB (SR9009, GSK4112, SR8278) or ROR (SR1078, SR1001) at 1-10 μM (15).

### 2.9 Functional Assays

#### 2.9.1 Shape change

Cells were pretreated as indicated, stimulated with 1-10 nM eotaxin-1/CC-chemokine ligand (CCL)11 for 4 min at 37°C, fixed, and analyzed by flow cytometry (25).

#### 2.9.2 *In vitro* migration assay

Eosinophils were pretreated and stimulated as indicated, chemotaxis was performed in a 48-well microBoyden chamber with 5 μm PVP-free polycarbonate filters for 60 minutes, and migrated cells were counted by flow cytometry (26).

#### 2.9.3 Apoptosis

Eosinophils were cultured in RPMI 1640 with 1% FBS, 1% Penicillin/Streptomycin, and IL-5 (50 pM). At 0, 3, and 22 hours, cells were stained with APC-Annexin-V and propidium iodide, and analyzed by flow cytometry, identifying early apoptotic cells (Annexin-V+/PI-) (27).

#### 2.9.4 Respiratory burst

Pretreated PMNL were stimulated with the respective chemoattractant in the presence of dihydrorhodamine-123 (Fisher Scientific). ROS production was indicated by an increase in fluorescent rhodamine by flow cytometry (28).

#### 2.9.5 Degranulation assay

PMNL were stained with CD16-PerCP-Cy5.5 to distinguish between CD16+ neutrophils and CD16-eosinophils and pretreated as indicated. Cells were mixed with cytochalasin B, stained with FITC-CD63 and analyzed by flow cytometry.

### 2.10 Phosphokinase Array

Eosinophils from three asthmatic or healthy donors were pooled and treated with SR1001 or DMSO for 3h. Protein was extracted and the Proteome Profiler Human Phospho-Kinase Array Kit (Bio-Techne) was performed according to the manufacturer’s instructions.

### 2.11 Phospho-Flow

SR1001- or DMSO-treated eosinophils were stimulated with eotaxin-1/CCL11 (1-10 nM) for 10 minutes, fixed, and stored overnight. Cells were stained with total-ERK and phospho-ERK antibodies (Cell Signaling) for 30 minutes and detected using a PE-conjugated donkey anti-rabbit secondary antibody.

### 2.12 Western Blot

Protein was extracted from eosinophils using RIPA buffer with protease inhibitors. Protein content was determined by BCA before gel loading and fast blotting. Membranes were blocked with 3% BSA in Tris buffered saline with Tween 20 (TBST) and incubated with primary antibodies against pERK, tERK, pJNK (Santa Cruz), and ß-actin (Cell Signaling). Detection was performed using the iBright system after applying secondary antibodies.

### 2.13 Animal experiment

#### 2.13.1 *In vivo* migration model

7-10-week-old Tg(CD2-Il5)5C2Ldt IL-5 transgenic mice (Tg) on the BALB/c background (29) of both sexes were treated i.p. with SR1001 (25 mg/ kg/ twice a day) or vehicle, 5 times in total. *In vivo* chemotaxis of eosinophils was induced by intranasal instillation of 5 μg eotaxin-2/CCL24. Bronchoalveolar lavage (BAL) fluid and blood was collected 4 hours afterwards. Immune cell composition was detected by flow cytometry using CD11b-PE-Cy7, CD11c-BV421, Ly6G-APC and Siglec-F-PE antibodies (BD Pharmingen) (30).

#### 2.13.2 Assessment of circadian behavior

The LabMaster system (TSE Systems) was utilized to analyze the impact of SR1001 on circadian locomotion, exploration, drinking, and feeding behavior of mice using transparent cages equipped with infrared beams and weight sensors. Data were recorded and analyzed using the LabMaster software (31).

#### 2.13.3 House Dust mite (HDM) model

8-12-week-old BALB/c mice of both sexes were challenged intranasally with 10 μg of HDM allergen (dissolved Acarizax SLIT-tablet) 4 times once a week. Thereafter, mice were treated i.p. with SR1001 (25 mg/kg, twice daily) or vehicle for a total of 5 doses. Airway hyperreactivity to methacholine was assessed with the FlexiVent platform (Scireq/Emka). BAL and blood were stained for leukocyte populations using the same protocol as described above, lungs were fixed with formalin and embedded in paraffin.

### 2.14 Periodic-Acid-Schiff (PAS) staining

Mouse lung sections of 5 μm were deparaffinized to perform a PAS staining. Slides were scanned with the Aperio slide scanner (Leica) and analyzed using ImageJ.

### 2.15 Statistical Analysis

Data are shown as mean ± SEM for n observations. Statistical analyses were performed using GraphPad Prism software 6.0 (La Jolla, CA; USA). To identify statistical outliers, Grubbs test was conducted and normal distribution was confirmed by Shapiro-Wilk test. Comparisons between groups were made using either a t-test or Mann-Whitney-U test. One-way or Two-way ANOVA followed by a post hoc test was applied as indicated. Probability values of p < 0.05 were considered statistically significant and are indicated as *p < 0.05; **p < 0.01; ***p < 0.001, and ****p < 0.0001.

## 3 Results

### 3.1 The MCC is disrupted in peripheral blood eosinophils from asthmatic patients

**Figure 1:**
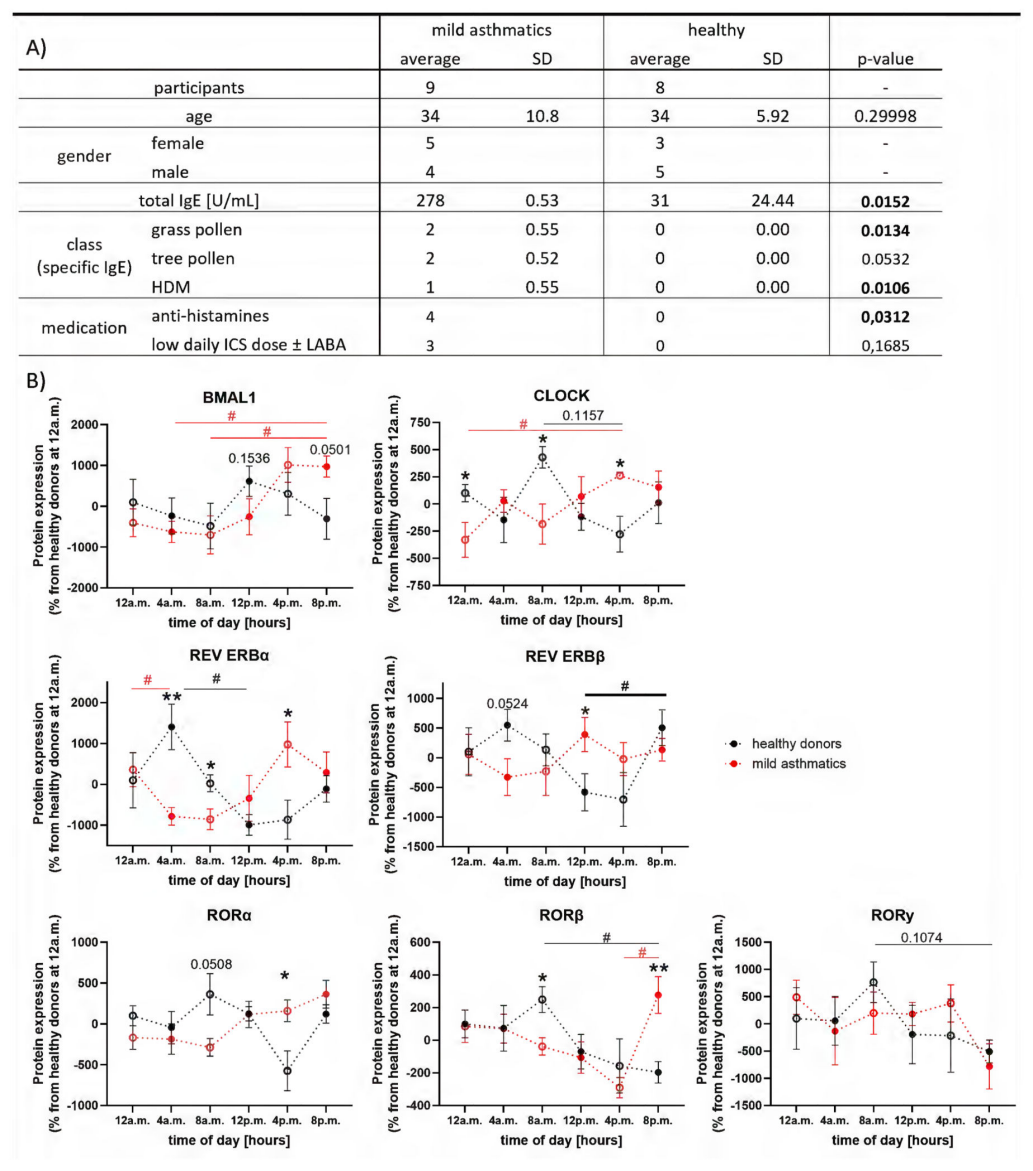
Differences in the oscillation pattern of clock proteins between eosinophils from asthmatic and healthy individuals. (A) Demographic table of healthy donors (n=8) and mild asthmatics (n=9). (B) Oscillating protein expression pattern of BMAL1, CLOCK, REV ERBs and RORs were detected in peripheral blood eosinophils. Samples analyzed immediately are represented by dots, while samples analyzed after 4 h of incubation are shown as circles. For statistical analyses, Z-scores were calculated and normalized to the mean of the healthy control group at 12 a.m. Group matched repeated Two-Way ANOVA, Tukey post hoc test. Comparison within the group is indicated with lines and hashtags (in matching color: healthy donors in black and mild asthmatics in red), while comparison between the two groups is indicated with stars. * and # represent p < 0.05, ** p < 0.01. BMAL1, Brain and muscle Arnt-like protein-1; CLOCK, circadian locomotor output cycles kaput; HDM, house dust mite; ICS, inhaled corticosteroids; IgE, Immunoglobulin E; ROR, retinoic acid receptor-related orphan receptor.

To validate the daily oscillation of clock proteins in eosinophils, we performed a flow cytometric 24-hour monitoring experiment as recently published (15). In brief, blood was collected from healthy donors and mild asthmatics recruited at the Medical University of Graz at 4 a.m., 12 p.m., and 8 p.m. Half of each sample was stained immediately, and the other half was stained after four hours, resulting in six time points per day. Eosinophils were gated by their FSC/SSC properties and respective surface markers (Figure S1). Participants were categorized as healthy or mild asthmatic according to GINA guidelines (22) and total/specific IgE levels (Figure 1A).

Our results clearly show that the clock proteins of the accessory loop are expressed in an oscillating manner in eosinophils and neutrophils from healthy donors (Figure 1B and S2). Interestingly, eosinophils and, to a lesser degree, neutrophils from mild asthmatics exhibited changes in period lengths and phase shifts in the oscillation of all clock proteins. In addition, a lower overall amplitude of oscillation was observed for CLOCK, REV ERBβ, RORα, and with a weaker extend for REV ERBα, compared to healthy donors. In healthy controls, BMAL1 peaked at 12 p.m. and showed its nadir at 8 a.m., while mild asthmatics exhibited a peak at 4 p.m. and a nadir at 8 a.m. CLOCK, the binding partner of BMAL1, peaked at 12 p.m. and reached its nadir at 4 p.m. in healthy donors, compared to 4 p.m. and 12 a.m. in mild asthmatics. The BMAL1 repressor REV-ERBα peaked at 4 a.m. and showed its nadir at 12 p.m. in healthy donors, while in mild asthmatics, it peaked at 4 p.m. and its nadir was observed at 8 a.m. For REV-ERBβ, peak and nadir were observed at 4 a.m. and 4 p.m. in healthy donors, and at 12 p.m. and 4 a.m. in mild asthmatics. The transcription activators RORα, β, and γ peaked at 8 a.m. in healthy donors and had their nadir at 4 p.m., 8 a.m., and 8 a.m., respectively. In mild asthmatics, RORα and β peaked at 8 p.m., while RORγ peaked at 12 a.m. Amplitude nadirs were observed at 8 a.m., 4 p.m., and 8 p.m., respectively.

**Figure 2:**
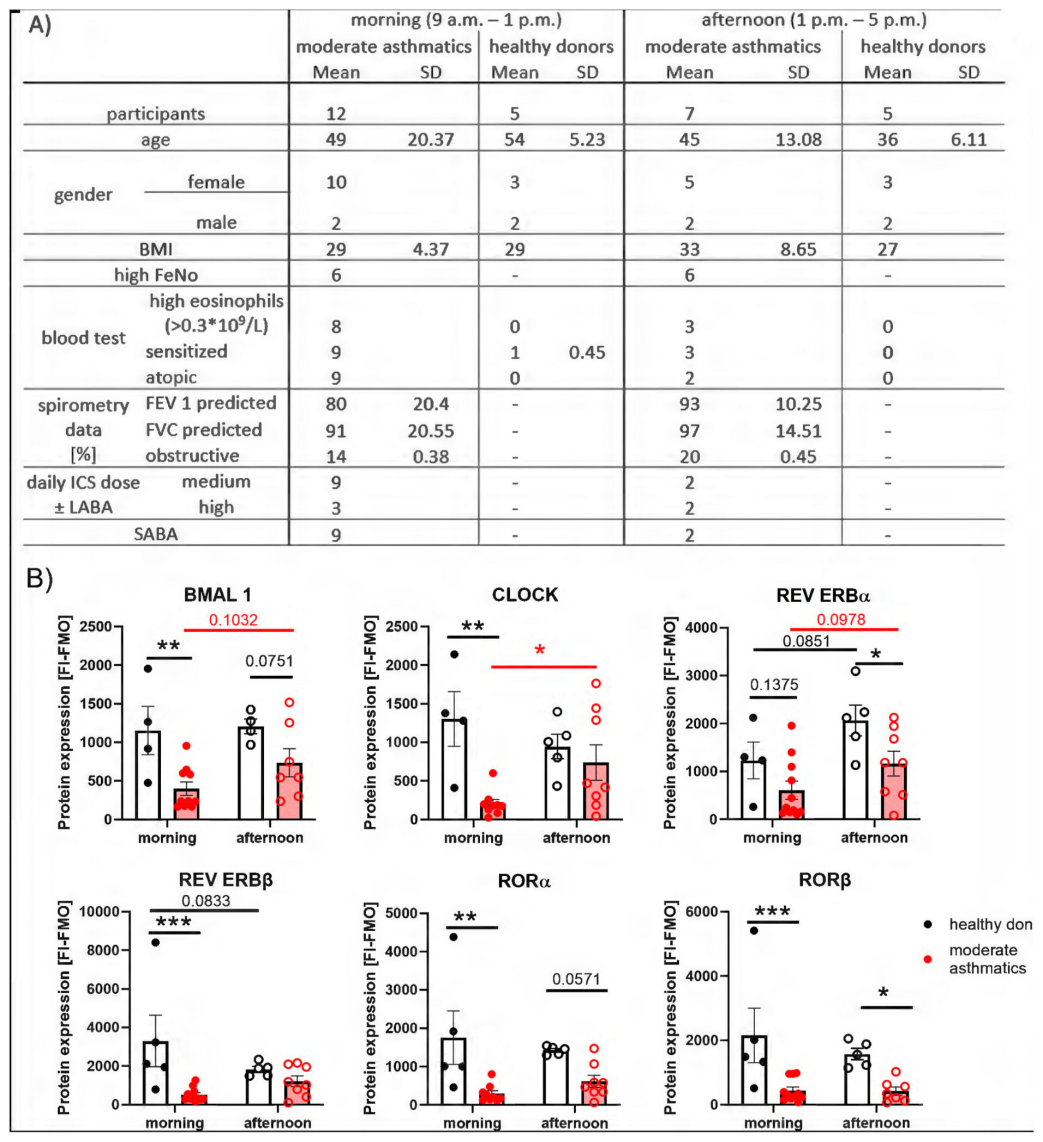
Clock protein expression is damped in eosinophils from patients with moderate asthma. (A) Demographic table of healthy donors (n=17) and moderate asthmatics (n=12). All participants were allocated to the morning (filled dots) or afternoon (empty circles) group depending on the time of evaluation. (B) Significant lower clock protein expression was observed in moderate asthmatics (red) compared to healthy donors (black) in both groups. Group matched, repeated Two-Way ANOVA, Tukey post hoc test, * p < 0.05, ** p < 0.01, *** p < 0.001. FeNO, fractional exhaled nitric oxide; ICS, inhaled corticosteroids; SABA, short-acting beta-2 agonists.

To underscore these results, we evaluated the MCC in another well-characterized cohort of moderate asthmatics recruited at the Wythenshawe Hospital in Manchester (Figure 2A). All analysis using cells from moderate asthmatic were performed within this cohort. Due to our small sample sizes, significant differences between morning and afternoon clock protein levels within the groups were only observed for REV ERBα/β in healthy donors and for CLOCK in moderate asthmatics. However, compared to healthy blood donors, significantly lower levels of all clock proteins, except for REV ERBα, were detected in eosinophils from moderate asthmatics in the morning. In addition, decreased REV ERBα and RORα levels were observed in the afternoon (Figure 2B). To compare the MCC of mild and moderate asthmatics from our two cohorts, protein levels of mild asthmatics at 4 p.m. were compared with the afternoon group of moderate asthmatics. The 8 a.m. and 12 p.m. time points were averaged and compared with the morning group (9 a.m. – 1 p.m.) of moderate asthmatics, as these time points most closely corresponded to the examination period of the moderate asthmatics. As shown in Figure S3, a decrease in eosinophil clock protein expression occurs in both mild and moderate asthmatics, but is more pronounced with higher severity of the disease, except for REV ERBα. Interestingly, in a small group of moderate asthmatics working shifts, opposing results were obtained for eosinophils with increased REV ERB and ROR expression compared to moderate asthmatics with conventional working schedule (Figure S4).

Similarly, neutrophil clock protein expression of moderate asthmatics, with the exception of REV ERBα, was significantly reduced in the morning, while lower levels of BMAL1, REV ERBα, and RORs were detected in the afternoon (Figure S5). Consistent with our previous research demonstrating reduced clock protein oscillation in monocytes from mild asthmatics (15), diminished clock protein levels, including decreased morning REV ERBα and RORβ expression, were detected in monocyte subsets of the moderate asthma cohort (Figure S6).

In addition, we were able to show that the MCC is detectable in tissue and sputum eosinophils from asthmatics (Figure S7).

### 3.2 Clock protein levels of peripheral blood eosinophils reflect asthma severity and the inflammatory environment

**Figure 3:**
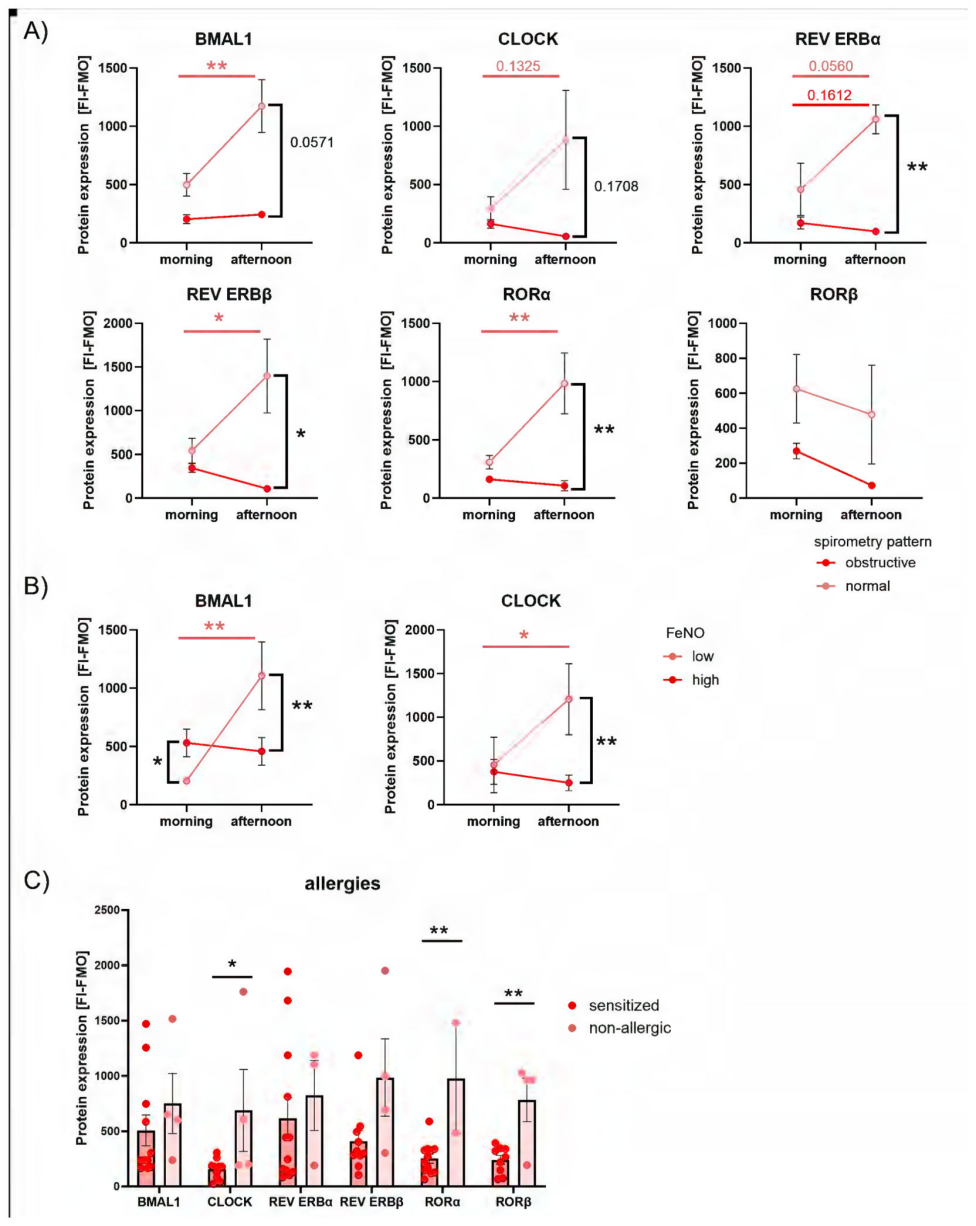
Low clock protein levels are linked to lung obstruction, allergy status and airway inflammation in patients with moderate asthma. (A) Clock protein expression of moderate asthmatics with obstructive (FEV1:FVC < 0.7) and normal spirometry pattern were compared (n≥5). (B) Patients with moderate asthma and high FeNO (threshold 50 ppb, n≥4) lack circadian variation in BMAL1 and CLOCK. (C) Sensitized asthmatics have significantly lower CLOCK, RORα and RORβ protein levels compared to non-allergic asthmatics. Group matched, repeated Two-Way ANOVA, Tukey post hoc test (A, B), unpaired t-test or Mann-Whitney-U test (C), * p < 0.05, ** p < 0.01.

Based on our data, we investigated whether clock protein levels of eosinophils from moderate asthmatics align with clinical parameters. FEV1 and FeNO, a non-invasive biomarker for eosinophilic airway inflammation, are known to show diurnal and seasonal variation (5, 32, 33). Accordingly, when evaluating the MCC in moderate asthmatics with normal and obstructive spirometry patterns (FEV1:FVC ratio < 0.7) (Figure 3A), and comparing FeNO^high^ (>50ppb) to FeNO^low^ patients (Figure 3B), patients were allocated to the morning or afternoon group based on the time of examination. Among patients with moderate obstructive asthma, we observed a decrease in BMAL1 and REV ERB in the afternoon, along with a loss of diurnal variation, compared to patients with better lung function (Figure 3A). Patients with high FeNO values displayed lower expression of BMAL1 and CLOCK in the afternoon, and lacked a normal time-of-day-dependent variation. Interestingly, patients allergic to at least one common allergen exhibited lower CLOCK and ROR levels, measured at a single time point (Figure 3C).

**Figure 4:**
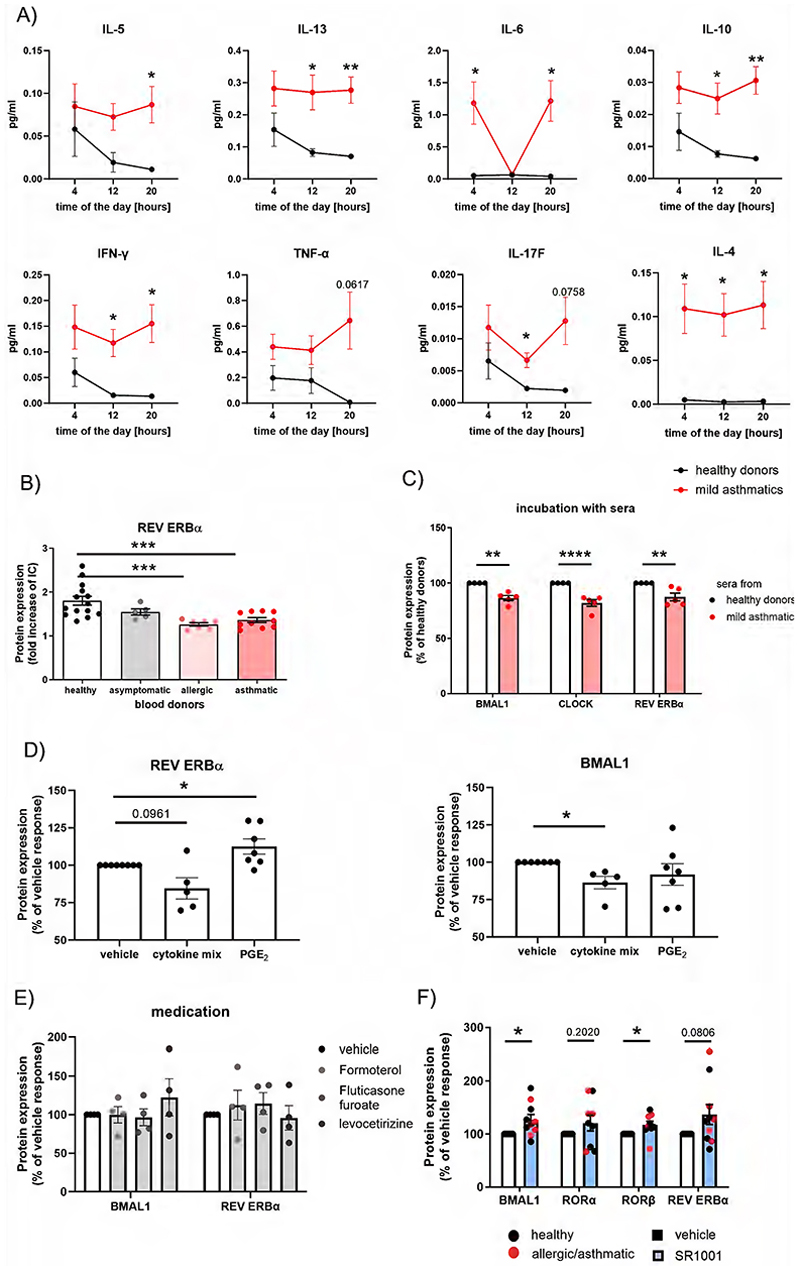
Bidirectional interplay between the circadian clock and the inflammatory mediators. (A) Circadian differences in circulating cytokine levels of patients with mild asthma (red) compared to healthy controls (black) determined by a multiplex assay (n≥8). (B) REV ERBα is decreased in eosinophils from mild asthmatics and allergic blood donors but not in asymptomatic sensitized volunteers (n≥5). Protein expression is calculated as fold increase over isotype control (=IC). (C) Stimulation of eosinophils from healthy donors with sera from mild asthmatics damped clock protein expression (n≥5). (D) Stimulation of PMNL from healthy donors with a cytokine mix or PGE_2_ alters REV ERBα expression. (n≥5). (E) Incubation with asthma/ allergy medication does not alter the peripheral clock of eosinophils (n=4). (F) The clock-modulating ligand SR1001 increases clock protein expression in non-allergic and allergic donors (n=10). Unpaired t-test or Mann-Whitney-U test (C,E), One-Way ANOVA (B,D) or Two-Way ANOVA (A), * p < 0.05, ** p < 0.01, *** p < 0.001, **** p < 0.0001.

Following from this, we further investigated the bidirectional interplay between the immune system and the circadian clock. As expected, increased serum levels of classical Th2 cytokines (34–36), IL-6 (37), IL-17-F, and TNF-α (38) were observed in mild asthmatics. A diurnal variation, with the highest levels measured in healthy donors at 4 a.m. was perceived. In asthmatics, cytokine levels are increased and additionally rise in the evening (8 p.m.), resulting in significant differences compared to healthy donors (Figure 4A). We also revealed an association between reduced REV ERBα expression and symptomatic allergies and/or asthma, again confirming the mutual influence of the immune system and the MCC (Figure 4B). To better understand this interaction, we exposed PMNL from healthy donors to sera from asthmatics which resulted in reduced REV ERBα in eosinophils (Figure 4C). Similarly, incubation with a cytokine cocktail suppressed REV ERBα and BMAL1 in eosinophils (Figure 4D), while PGE_2_, a bronchoprotective prostaglandin that inhibits eosinophil effector function (39), upregulated REV ERBα. Some asthma patients were under medication at the time of analysis, while others were not. To investigate the direct effect of betamimetics, corticosteroids and antihistamines on clock protein levels, eosinophils were incubated with formoterol, fluticasone and levocetirizine for three hours. Interestingly, neither formoterol nor fluticasone and levocetirizine directly affected the accessory loop, at least not at concentrations corresponding to systemic concentrations observed after inhalation or oral intake (40–43) (Figure 4E). Having observed a disease-related decline in clock protein expression, which cannot be reversed by current asthma therapeutics, we next investigated whether synthetic clock-modulating ligands are able to restore the MCC in eosinophils. Indeed, treatment of cell from healthy and allergic/asthmatic donors with an inverse ROR agonist (SR1001) for 3 hours increased ROR as well as BMAL1 and REV ERBα protein levels (Figure 4F). Again, the observed effects indicating a mutual interaction between the MCC and the immune system was more pronounced in eosinophils than in neutrophils (Figure S8).

### 3.3 SR1001 blocks CCR3 and EGFR-signaling

**Figure 5:**
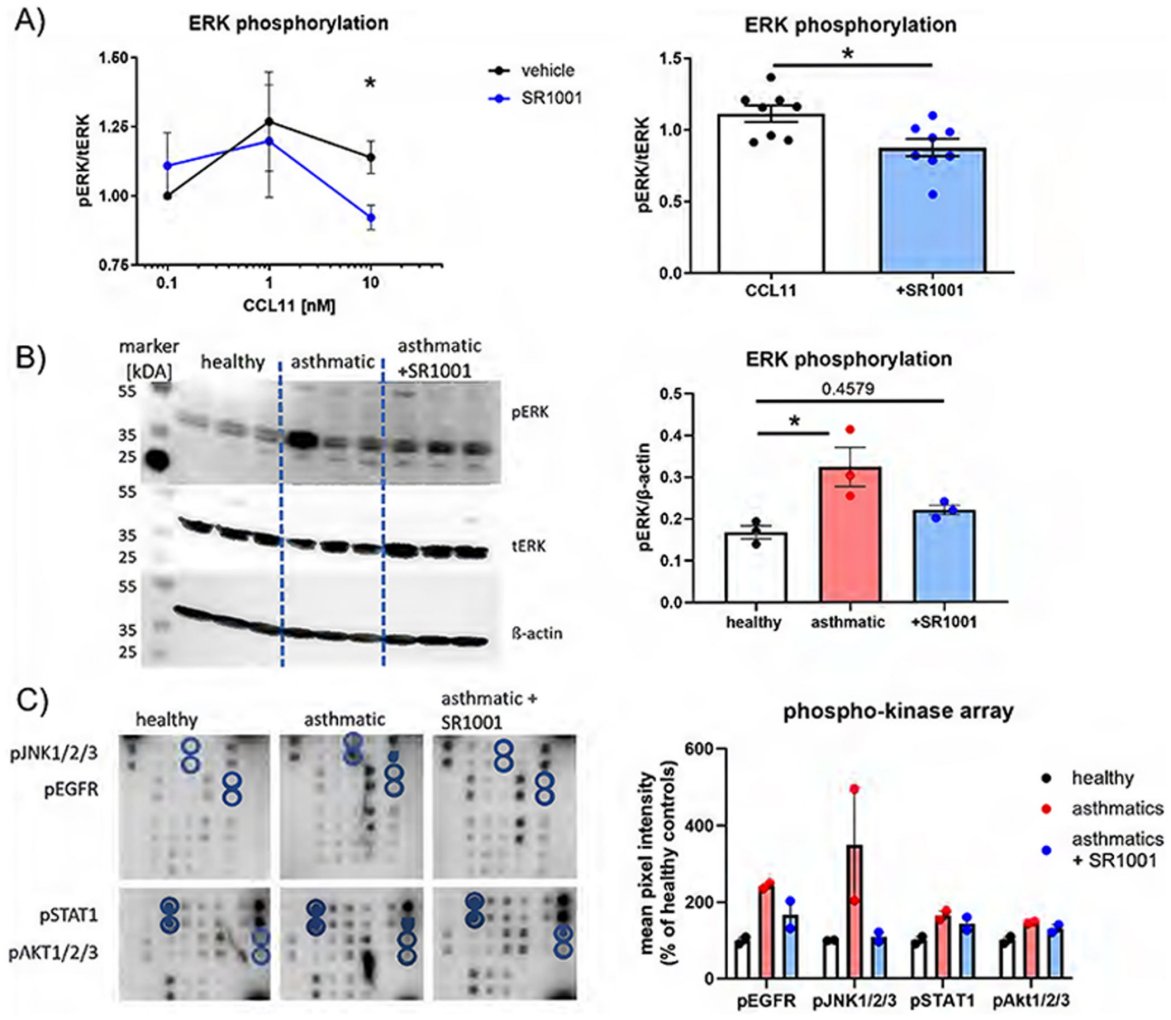
The inverse ROR agonist SR1001 blocks CCR3 and EGFR signaling. (A) ERK phosphorylation was detected in a Phospho-Flow Assay in response to eotaxin-1/CCL11. Data are presented as the ratio of phosphorylated ERK to total ERK (n=8). (B) Western blotting confirmed that SR1001 reduces the increased ERK phosphorylation in asthmatics (duplicates measured from n=3). (C) Eosinophils from three healthy or asthmatic donors were pooled to achieve a total of 4.5*10^6^ eosinophils per group. Cells were treated with either 10 μM SR1001 or control DMSO for 3 hours. Afterwards a phosphokinase array was performed. T-test, One-Way ANOVA, Tukey post hoc test, * p < 0.05. CCL11, CC-chemokine ligand 11; CCR3, C-C chemokine receptor type 3; EGFR, epidermal growth factor receptor; ROR, retinoic acid receptor-related orphan receptor.

Recent reports have indicated that BMAL1 upregulation leads to decreased ERK phosphorylation (44). Since ERK signaling is a common pathway for many pro-inflammatory mediators such as EGF and eotaxin-1/CCL11, a CCR3 ligand, and known to be increased in eosinophils from asthmatics (45), we aimed to explore the impact of SR1001 on these signaling cascades. In a Phospho-Flow-assay and by Western blotting we observed that SR1001 blocks both eotaxin-1/CCL11-induced ERK phosphorylation in healthy donors as well as increased phospho-ERK levels in mild asthmatics (Figure 5A-B). A phosphokinase array further revealed that opposed to healthy controls, phosphorylation of EGFR, c-Jun-N-terminal kinase (JNK1/2/3), signal transducer and activator of transcription (STAT)1 and Akt1/2 is increased in eosinophils from asthmatics and again declined after SR1001 treatment (Figure 5C).

### 3.4 SR1001 mediates anti-inflammatory effects *in vitro* and *in vivo*

**Figure 6:**
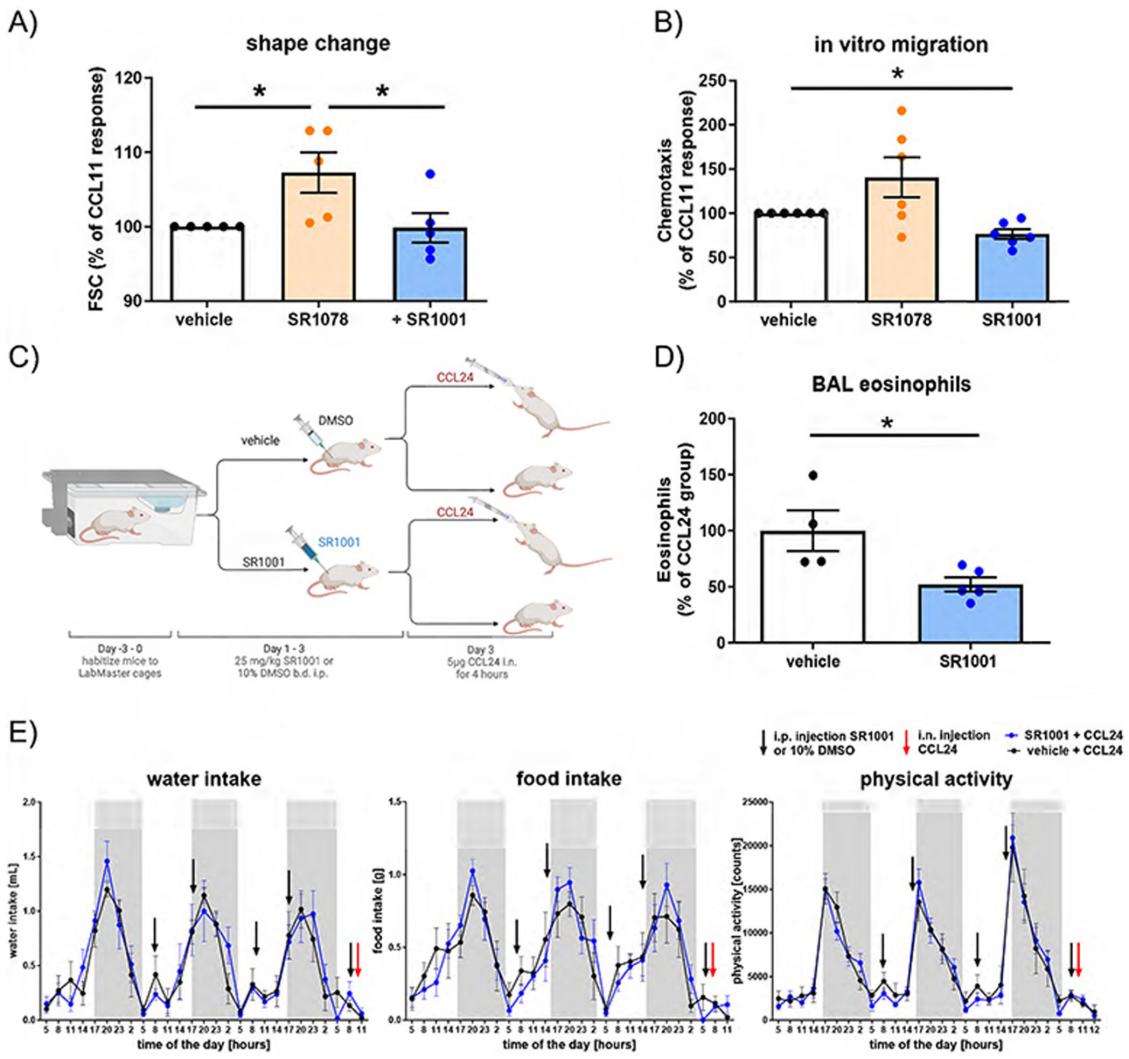
SR1001 inhibits eosinophil migration *in vitro* and *in vivo* without affecting circadian behavior. PMNL (A) or isolated eosinophils (B) were pretreated with 10 μM SR1001 and/or treated with 10 μM SR1078. (A) After the treatment, a shape change assay towards eotaxin-1/CCL11 was performed (n=5). (B) Chemotaxis towards eotaxin-1/CCL11 was performed with isolated eosinophils from allergic donors (n≥4). (C) *In vivo* migration model: After a 3-day acclimatization period in the LabMaster cages, mice were treated 5 times with 25 mg/kg SR1001 i.p. (black arrow). Four hours after the last dose, mice were instilled with 5 μg eotaxin-2/CCL24 i.n. (red arrow). (D) BAL was collected and evaluated by flow cytometry (n≥4). (E) Circadian behavior (drinking, eating rhythm and physical activity) was continuously recorded by the automated home cage phenotyping LabMaster system (n≥5). T-test (D), matched One-Way (A, B) or group matched, repeated Two-Way ANOVA, Tukey post hoc test (E), * p < 0.05. BAL, bronchoalveolar lavage; CCL11, CC-chemokine ligand 11; i.n., intranasal; PMNL, polymorphonuclear leukocytes.

Investigating the impact of synthetic ROR ligands on eosinophil effector function, we observed that the ROR agonist SR1078 induced reactive oxygen species (ROS) production, degranulation (Figure S9), and cytoskeleton rearrangement (Figure 6A). Pretreatment with SR1001 not only diminished these responses, but also inhibited the migration of eosinophils from allergic donors (Figure 6B). Once more, SR1001 was more effective in eosinophils than in neutrophils (Figure S10).

Consistent with previous studies (15, 46), the REV-ERB agonists GSK4112 and SR9009 not only increased REV-ERB levels and mediated certain anti-inflammatory effects (Figure S11 A-C, Figure 12 A-B), but also promoted pro-inflammatory responses such as increased ROS production in eosinophils and neutrophils, which was partly REV ERB independent (Figure S11D, S12C). Therefore, for further *in vivo* studies, only SR1001 was used. In an *in vivo* migration model, pretreatment of IL-5 transgenic mice with SR1001 before instillation of eotaxin-2/CCL24 significantly reduced the recruitment of eosinophils into the airways (Figure 6C-D). To ascertain that targeting the MCC with SR1001 does not affect the animals’ rhythmic biological behavior, we utilized LabMaster cages. Similar to previous results from wild type mice (15), no changes in drinking behavior, eating rhythm or rhythm of physical activity was noticed during the experiment (Figure 6E).

**Figure 7:**
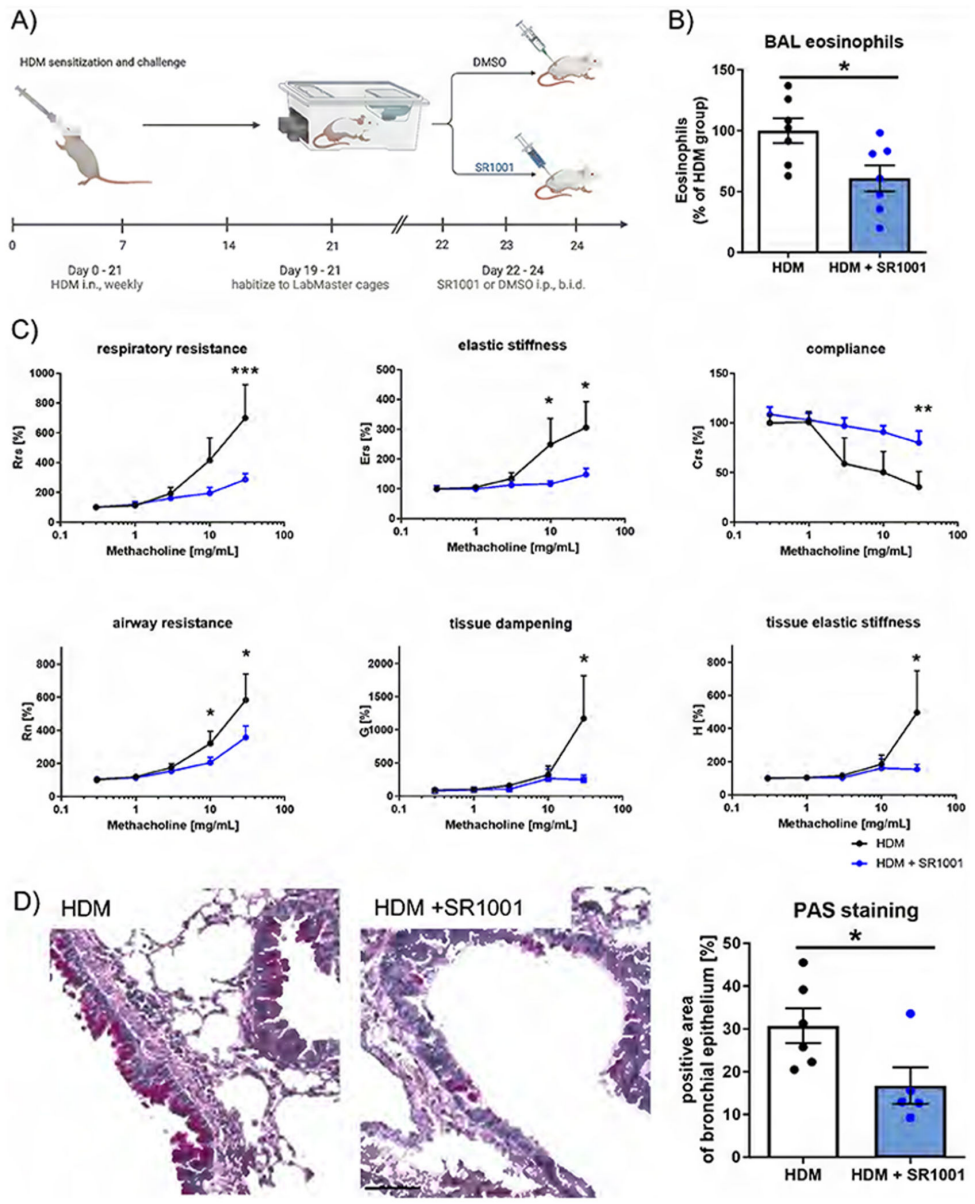
SR1001 promotes anti-inflammatory and bronchoprotective effects in HDM-induced airway inflammation. (A) Mice were treated i.n. with HDM extract (10 μg) 4 times once a week. Starting from day 22, after a 3-day acclimatization period in the LabMaster cages, mice were treated 5 times with 25 mg/kg SR1001 i.p. After the last SR1001 injection on day 24, BAL was collected. (B) To evaluate the effect of SR1001 on airway inflammation, CD11c^low^ Siglec-F^high^ eosinophils in the BAL were determined by flow cytometry (n≥7). (C) Effects on airway hyperreactivity in response to methacholine were recorded by using the FlexiVent platform (n≥7). (D) Mucus production was evaluated histologically by PAS staining. Representative images are shown (scale bar 100 μm, n≥5). T-test (B, D), matched, repeated Two-Way ANOVA (C), Šidák post hoc test. * p < 0.05, ** p < 0.01, *** p < 0.001. Crs, Compliance of the respiratory system; Ers, Elastance of the respiratory system; G, Tissue damping; H, Tissue elastance; HDM, house dust mite; i.n., intranasal; PAS, periodic acid Schiff; Rn, Newtonian (airway) resistance; Rrs, Resistance of the respiratory system.

To further validate blocking ROR as a therapeutic approach, an HDM-induced lung inflammation model was used. Mice were challenged with HDM to induce lung inflammation, followed by five therapeutic injections with SR1001 (Figure 7A). Consistent with our previous data, SR1001 significantly decreased the proportion of eosinophils but not of neutrophils in the BAL (Figure 7B, S10B). Additionally, treated mice exhibited improved lung function indicated by decreased resistance and improved compliance along with other parameters (Figure 7C). Furthermore, compared to the control group, lungs from SR1001-treated mice showed significantly reduced mucus plugging in the PAS staining (Figure 7D).

## 4 Discussion

In this study, we employed a translational approach to identify an active yet unbalanced MCC in eosinophils from asthmatic patients. By resetting the MCC, clock-modulating ligands could represent a novel anti-inflammatory treatment option, as they ameliorate airway inflammation, reduce mucus production, and improve lung function without disturbing the daily biorhythm.

Although the oscillation of clock genes in the lung is established (47–49), their role within the immune system and under inflammatory conditions is less understood. Our lab recently demonstrated that the MCC is disrupted in monocytes and macrophages during allergic inflammation (15). Similarly, Chen et al. reported reduced clock gene expression in PBMCs from asthma patients (16). However, we are the first to observe an overall reduction of clock proteins in eosinophils, dependent on disease severity. Noteworthy, these effects were not specific to eosinophils but were also found to a lesser extent in neutrophils and monocytes. Our results align with *in vivo* studies showing that *Bmal1* or *Rev Erb* ablation worsens inflammatory diseases in mice (20, 50–52). We are also the first to link this disrupted MCC to clinical parameters: reduced eosinophil BMAL1 and REV ERBα/β appear sensitive to airflow obstruction, low CLOCK and RORα/β are associated with allergic asthma, and a lack of circadian variation of BMAL1 and CLOCK was observed in patients with high FeNO level, indicating active eosinophilic asthma. Although our analyses provide significant results, we are aware that our findings still need to be validated in a larger cohort. Moreover, for the 24-h monitoring experiment, samples were stained immediately or after 4 hours of incubation at 37°C to achieve 6 time points of evaluation. Preliminary experiments from our group as well as previous studies confirm that the MCC remains active *in vitro/ex vivo* even for several days (15, 23), however, we cannot rule out the possibility that influences such as *in vivo* fluctuations in circadian hormones or cytokine levels are not correctly reflected by incubated time points.

Epidemiologic studies suggest that female gender and obesity are important risk factors for severe asthma in adulthood (53, 54), which is also reflected in the demographic differences between our cohorts with mild and moderate asthma. However, no differences in clock protein expression related to sex, age or body mass index were found. Importantly, in a small group of shift workers with moderate asthma no clear MCC pattern could be identified, while significant differences in REV ERBα/β and RORα/β were observed compared to patients with conventional working hours. It is known that shift work disrupts the circadian system and, thus, is a risk factor for chronic pulmonary diseases (55). Accordingly, Durrington et al. revealed a 23% higher prevalence of asthma and significantly lower lung function in night shift workers compared to day workers (19).

Another evidence of the bidirectional interaction between the circadian clock and the immune system is the daily fluctuation in circulating cytokines. Our study reveals increasing circulating cytokine levels in the early morning hours in healthy donors, while in asthmatics, cytokine levels are generally increased and further rise in the evening. This observation may contribute to our understanding of nocturnal asthma attacks, which are known to be driven by cytokines such as TNF-α and IL-6 (56). Tang et al. found that the increased IL-6 levels in patients with nocturnal asthma compared to those without nocturnal symptoms are associated with blocked BMAL1/FOXA2 signaling in airway epithelial cells (57). Likewise, we confirmed that the disruption of the MCC in eosinophils is driven by systemically increased inflammatory mediators. Already short-term exposure of donor cells with sera from asthmatics or a cytokine cocktail, decreased clock protein expression to levels of asthmatics. In contrast, bronchoprotective PGE_2_, known to impede eosinophil function (58), increased the anti-inflammatory repressor REV ERBα. This observation is in accordance with Tsuchiya et al. who demonstrated that PGE_2_ acts as an clock-resetting agent in cultured fibroblasts and peripheral tissues (59). Since several asthma patients used inhaled corticosteroids, betamimetics and anti-histamines at the time of evaluation, we examined their direct effects on circadian clock protein levels. A three-hour treatment with fluticasone (41), formoterol (40) or levocetirizine (42, 43) at concentrations corresponding to plasma levels observed after inhalation or oral administration had no impact on the accessory loop in eosinophils. These results confirm that asthma medication has no immediate effect on the molecular clock; however, long-term effects cannot be ruled out. Intriguingly, the ROR inverse agonist SR1001 reset the MCC by increasing ROR, BMAL1 and REV ERBα levels in healthy and asthmatic/allergic donors to a similar extend. However, the underlying mechanism still needs to be clarified.

Similar to RORγ, besides being a transcription activator of BMAL1, RORα drives Th17 signature genes and, thus, targeting RORα has beneficial effects in autoimmune diseases (60, 61). In addition to its clock-modulating properties, we and others revealed the anti-inflammatory potential of SR1001 (62–65) for instance by inhibiting secretion of macrophage inflammatory protein from M1 macrophages, an eosinophil chemoattractant driving airway inflammation, as well as the infiltration of pro-inflammatory monocyte-derived alveolar macrophages into the airways of HDM-challenged mice (66). Here we demonstrate that SR1001 also inhibits eosinophil recruitment without affecting the circadian rhythmicity of the animals. Further, in the HDM model, SR1001 reduced the infiltration of eosinophils into the airways, improved lung function, and decreased mucus production in the lungs. Since SR1001 targets not only the MCC but also transcription factors involved in T cell differentiation, the observed anti-inflammatory and bronchoprotective effects cannot be attributed solely to the resetting of MCC in eosinophils.

Interestingly, a direct link between BMAL1 upregulation and reduced ERK phosphorylation, a crucial downstream signaling pathway of the eosinophil chemoattractant CCL11, has been described previously (44). Durrington et al. revealed a circadian variation of CCL11 in sputum, peaking at 4 a.m. and coinciding with the peak in eosinophil influx, which is associated with a higher risk of severe asthma attacks (3). Here we demonstrated that SR1001 prevents CCL11/CCR3-induced ERK phosphorylation in eosinophils, possibly by increasing BMAL1 and thereby impeding this crucial pathomechanism of asthma. However, we cannot exclude the possibility that SR1001 also affects the phosphorylation of clock proteins, a translocation and degradation signal, thereby increasing their protein content. To clarify this crucial mechanism, however, further research is required.

SR1001 also reduced ERK phosphorylation in eosinophils from asthmatics almost to resting-state levels of healthy donors. Additionally, dysregulation of EGFR and its downstream pathways contribute to epithelial barrier dysfunction, mucus production, and airway inflammation (67). Since SR1001 blocked exaggerated EGFR activation in patients with asthma by suppressing autophosphorylation and JNK/STAT/Akt activation, we propose a cross-talk between the MCC and EGFR signaling. To our knowledge, so far only one publication suggested a similar interaction between RORγ and EGFR/ERK signaling (68). Moreover, a CCR3-dependent activation of EGFR has been described in bronchial epithelial cells (69). These results underline the importance of CCR3, EGFR, and their downstream pathways in the pathogenesis of asthma. Consequently, we hypothesize that SR1001 counteracts eosinophil hyperactivation possibly via BMAL1-induced prevention of ERK phosphorylation, CCR3 and EGFR signaling.

In conclusion, we demonstrate that the MCC oscillates at the protein level in human eosinophils, but exhibits marked inflammation-related changes and a general attenuation in asthma patients. Moreover, the MCC of eosinophils reflects disease severity, airflow obstruction and allergy status of the patients. Targeting the nuclear receptor family ROR resets the MCC in eosinophils and promotes anti-inflammatory and bronchoprotective effects without disrupting the circadian biorhythm. Thus, clock-modulating ligands might represent a promising steroid-free anti-inflammatory treatment option for chronic inflammatory airway diseases such as asthma.

## Supplementary Material

Supplemental Material: https://doi.org/10.6084/m9.figshare.28616447

Supplemental material and figures

## Data Availability

Data will be made available upon reasonable request.

## Brief Summary

Our findings highlight the role of the circadian system as an immunomodulatory regulator, biomarker, and therapeutic target in chronic inflammatory diseases. The observed inflammation-driven downregulation of the molecular circadian clock may also represent a key mechanism that triggers the switch from homeostatic to pro-inflammatory eosinophils. Furthermore, we demonstrate for the first time that pharmacologic inhibition of ROR resets the molecular circadian clock and induces anti-inflammatory and lung-protective effects without disrupting circadian rhythms.

## Abbreviation

BAL: Bronchoalveolar lavage
BMAL 1: Brain and muscle Arnt-like protein-1
CCLL: CC-chemokine ligand
CCR3: C-C chemokine receptor type 3
CLOCK: Circadian locomotor output cycles kaput
EGFR: Epidermal growth factor receptor
FeNO: Fractional exhaled nitric oxide
FEV1: Forced expiratory volume in 1 second
FSC: Forward scatter
HDM: House dust mite
IgE: Immunoglobulin E
IL: Interleukin
JNK: Jun-N-terminal kinase
MCC: Molecular circadian clock
PMNL: Polymorphonuclear leukocyte
ROR: retinoic acid receptor-related-related orphan receptor
ROS: reactive oxygen species
STAT1: Signal transducer and activator of transcription
SSC: Side scatter
TNF-α: Tumor necrosis factor α
