# Supplementary material for: The molecular circadian clock of eosinophils: A potential therapeutic target for asthma
Source: Am J Physiol Cell Physiol. Author manuscript; Available in PMC 2025 Jun 21. (PMC7617784; doi:10.1152/ajpcell.00149.2025)
Supplement: Supplemental material and figures [file EMS204166-supplement-Supplemental_material_and_figures.pdf]

Supplemental Material and Figures

Ethical Approval

Experiments with human material were authorized by the Institutional Review Board of the Medical University of Graz (blood: EK 17–291 ex 05/06; biopsies: EK Nr: 1170/2024) and the Manchester Allergy, Respiratory and Thoracic Surgery Biobank (ManARTS) Rec: 20/NW/0302, IRAS: 285126 (North West - Haydock Research Ethics Committee) through Manchester University NHS Foundation Trust (M2023-133), and were performed with the informed consent of all donors. Blood donors were categorized as healthy, allergic and/or asthmatic based on a clinical diagnosis from external respiratory physicians following the GINA guidelines, IgE levels, and reported symptoms. Total (≥100 kU/l) and specific IgE (class 1: 0,35-0,69 kU/l; class 2: 0,7-3,49 kU/l; class 3: 3,5-17,4 kU/l; class 4: 17,5-49,9 kU/l: class 5: 50-100; class 6 >100) was measured at the Department of Dermatology and Venerology of the Medical University of Graz.

Animal experiments were approved by the Austrian Federal Ministry of Science and Research's Animal Ethics Committee, adhering to the European Community's Council Directive (BMWF-2022-0.626.093).

24-h monitoring experiment

To validate the daily oscillation of clock proteins in peripheral blood leukocytes, we performed a flow cytometric 24-hour monitoring experiment. In brief, blood was collected at 4 a.m., 12 p.m., and 8 p.m. Half of each sample was stained immediately, and the other half was stained after four hours of incubation at 37°C, resulting in six time points per day. Flow cytometric staining, gating and analysis is described below. It is generally known and also confirmed by our previous study and preliminary experiments, that the MCC remains active *in vitro/ex vivo* for several hours.

Whole blood staining

In whole blood samples leukocyte populations were stained with CD3-APC-Cy7 (Biolegend, California, USA, 317342), CD14-BV421 (Biolegend, 301830) and CD16-PerCP-Cy5.5 (Biolegend, 301828). Eosinophils were distinguished from neutrophils by autofluorescence using BV450 channel. Cells were fixed and permeabilized with FIX and PERM (Nordic-MUbio, Netherlands, GAS-002). Samples were then blocked with human TruStain FcX™ Fc Receptor Blocking Solution (Biolegend, 422302) and clock proteins were stained intracellularly using the following primary antibodies: NR1D1 (Abcam, United Kingdom, ab174309), REV ERB beta (Novusbio, Colorado, USA, NBP2-19576), BMAL1 (Novusbio, NB100-2288), CLOCK (Mybiosource, California, USA, MBS4750976), ROR alpha (ThermoFisher Scientific, Massachusetts, USA, PA1-812) ROR beta (Novusbio, NBP1-82532) and ROR gamma-PE (R&D

Systems, Minnesota, USA, IC6006P). The secondary donkey anti rabbit -PE antibody (Biolegend, 406421) was used for detection. Samples were measured on a BD FACSCanto II flow cytometer and analyzed as positive FI-FMO using FlowJo 10.8.1.

Intracellular staining of isolated cells

Cells were fixed and permeabilized with FIX and PERM (Nordic-MUbio, GAS-002). Thereafter, the samples were blocked with human TruStain FcX™ Fc Receptor Blocking Solution (Biolegend, 422302) and circadian proteins were stained intracellularly using primary antibodies NR1D1 (Abcam, ab174309), BMAL1 (Novusbio, NB100-2288) and ROR beta (Novusbio, NBP1-82532). The secondary donkey anti rabbit -PE (Biolegend, 406421) antibody was used for detection. Samples were measured by flow cytometry, analyzed by FlowJo 10.8.1. and presented as fold increase over the isotype control signal or positive fluorescence-minus-one (FI-FMO) signal. Controls included unstained sample, FMOs, secondary antibody only and isotype control.

Isolation of human peripheral blood polymorphonuclear leukocyte (PMNL) and eosinophils

Human peripheral blood PMNL and eosinophils were isolated from healthy donors independent of sex and age using dextran sedimentation, followed by density gradient centrifugation. Purification of eosinophils was performed with the Eosinophil Isolation Kit from Miltenyi Biotec according to the manufacturer's protocol.

Cell isolation from sputum

The cell fraction was isolated from the saliva portion of the sputum sample. Based on the sample's weight, four times its volume of 1:10 diluted DTT (D0632-5g, Sigma) in PBS (P4417, Sigma) was added. The sample was briefly vortexed and then incubated on a tube roller for up to 30 minutes at room temperature. Subsequently, the sample was filtered and centrifuged (10 min, 320xg, at 4°C). The isolated cells were washed with PBS and then subjected to the whole blood staining and intracellular staining protocols as described above.

Immunofluorescent staining

Biopsies from asthmatic patients and non-tumorous human lung samples underwent deparaffinization followed by a heat-induced antigen retrieval in sodium citrate buffer (pH 6). Unspecific binding was blocked with 4% BSA (Sigma, A7906) and 10% goat serum (Sigma, G9023) in PBS for 2 h at room temperature. Primary antibodies against BMAL1 (Novusbio, NB100-2288, 1:100 dilution) and EPX (clone MM25-82.2; 5 μg/ml; kindly donated by Dr. Elizabeth Jacobsen) were incubated overnight. Sections were incubated with Cy3-labeled donkey anti-rabbit and Alexa Fluor-

488-labelled goat anti-mouse secondary antibodies (both from ThermoFisher Scientific, A17692 and A11001, 1:500) for 2 h on the next day. Nuclear counterstaining was performed with the TrueVIEW® Autofluorescence Quenching Kit containing DAPI. All images were acquired using constant laser settings with a Nikon A1+ confocal microscope. Constant laser settings were applied for all image acquisitions. Control slides were stained with secondary antibodies only.

Cytokine Multiplex Array

Twelve human cytokines, including IL-2, 4, 5, 6, 9, 10, 13, 17A, 17F, 22, IFN-γ and TNF-α were simultaneously determined in serum samples from the monitoring experiment by using the Human Th Cytokine Panel from Biolegend (741028) according to the manufacturer's instructions.

Inflammatory mediators

Isolated PMNL were stimulated with either PGE_2 (100 nM; Cayman Europe, 14010) or a cytokine cocktail for 3h at 37°C. Based on the results from the cytokine multiplex array, a mixture of IL-4 (0,1 pg/ml; Immunotools, 1134004), IL-5 (0,1 pg/ml; Peprotech, 200-05-10UG), IL-6 (1,5 pg/ml; Peprotech, 200-06), IL-10 (0,03 pg/ml, Immunotools, 11340103), IL-13 (0,3 pg/ml, Biolegend, 571102) and IFN-y (0,15 pg/ml, Immunotools, 1134353) was prepared. Mediators were diluted in RPMI 1640 (Fisher Scientific, 21875091) supplemented with 1% FBS, 1% Penicillin/Streptomycin (Thermo Fisher, P06-07100).

Asthma/ allergy medication

Isolated human PMNL were treated with formoterol (50 pM; Selleckchem, S2020) and fluticasone furoate (500 pM; Selleckchem, S6476) for 3h at 37°C.

Clock-modulating agonists

Human PMNL or isolated eosinophils were pretreated with REV ERB or ROR agonists for 3h at 37°C. For all experiments a concentration of 1-10 µM of SR9009 (Sigma Aldrich, 1379686-30-2) and GSK4112 (Sigma Aldrich G0673), 10 µM of SR8278 and SR1001 (MedChemExpress, 1334106-03-0) as well as 5-10 µM of SR1078 (MedChemExpress, 1246525-60-9) was used.

Shape change

After agonist treatment, the cells were stimulated with eotaxin-1/CCL11 (Immunotools, 11343215; eosinophils) or IL-8 (Immunotools, 11349084; neutrophils) for 4 minutes at 37°C and fixed on ice. Shape change was detected by flow cytometry as an increase in FSC, analyzed using FlowJo 10.8.1, and expressed as percentage of the vehicle control.

Chemotaxis

Chemotaxis assays were performed in purified eosinophils in response to eotaxin-1/CCL11 (Immunotools, 11343215) or in PMNL in response to IL-8 (Immunotools, 11349084) for 60 min by using a 48-well microBoyden chemotaxis chamber. Chambers were equipped with PVP-free polycarbonate filters with a pore size of 5 μm. Migrated cells were collected from the bottom wells, fixed, enumerated by flow cytometry (acquisition: 30 sec at medium flow rate), and analyzed with FlowJo 10.8.1.

Apoptosis

Purified eosinophils were cultured in RPMI 1640 (Fisher Scientific, 21875091) supplemented with 1% FBS, 1% Penicillin/Streptomycin (Thermo Fisher, P06-07100), and 50 pM IL-5 (Peprotech, 200-05-10UG). After 0h, 3h and 22h, cells were stained with APC-Annexin-V (Biolegend, 640941) in the dark for 20 min at 4°C, followed by Propidium iodide (1:50, Biolegend 421301) in the dark for 1 min at room temperature. Samples were immediately measured by flow cytometry (acquisition: 1 min, at medium flow rate) and total cell numbers gated on a forward scatter/side scatter plot and the percentage of live cells (Annexin-V negative/propidium iodide negative), early apoptotic cells (Annexin-V positive/propidium iodide negative), late apoptotic cells (Annexin-V positive/propidium iodide positive) and necrotic cells (Annexin-V negative/propidium iodide positive) were recorded. Samples were further analyzed using the FlowJo 10.8.1 software.

Respiratory burst

Purified human PMNL were pre-treated and stimulated with serial dilutions of the respective chemoattractant in the presence of 1 μM dihydrorhodamine-123 (Sigma Aldrich, D1054) for 20 min at 37 °C as described. Production of reactive oxygen species (ROS) was quantified by flow cytometry as the increase of fluorescence due to oxidation of nonfluorescent dihydrorhodamine-123 into fluorescent rhodamine-123 and analysed by FlowJo 10.7.1 software. Responses were expressed as geometric mean fluorescence intensity (MFI).

Degranulation assay

PMNL were stained with CD16-PerCP-Cy5.5 (Biolegend, 301828) to separate CD16+ neutrophils from CD16- eosinophils. Further, cells were mixed with 5 μg/ml cytochalasin B (Sigma Aldrich, C6762), stained with CD63-FITC (Biolegend, 353006), measured by flow cytometry, and analyzed using the FlowJo 10.8.1 software.

Phosphokinase Array

The proteome profile phosphokinase array from Bio-Techne (ARY003C) was performed according to the manufacturer's instructions. In brief, isolated eosinophils from three asthmatic donors or three healthy donors were pooled together. Cells were divided again and treated with 10 µM SR1001 or DMSO as a control for 3 hours. After a washing step protein was extracted from the cells. Diluted cell lysates were incubated overnight with the membrane. Capture and control antibodies were spotted in duplicate on nitrocellulose membranes. The array was washed, incubated with biotinylated detection antibodies, and then treated with Streptavidin-HRP and chemiluminescent detection reagents. Signals at each capture spot corresponded to the amount of phosphorylated protein bound.

Phospho-Flow

Eosinophils were treated with SR1001, SR1078 or DMSO as a control for 3 hours. Afterwards, cells were stimulated with eotaxin-1/CCL11 (1-10 nM) for 10 min at 37°C. Eosinophils were fixed and stored in the fridge overnight. On the next day the cells were stained for total ERK (1:400 dilution, Cell signaling, 4695S) and phospho-ERK (1:400 dilution, Cell signaling 90101S) for 30 min at 4°C, followed by a PE-conjugated donkey anti-rabbit (1:500 dilution, Biolegend 406421) secondary antibody for 30 min at 4°C. Samples were measured by flow cytometry and analyzed by FlowJo 10.8.1 software.

Western blot

Protein was extracted from isolated eosinophils using RIPA lysate buffer (Thermo Fisher, 89900) supplemented with three-time protease inhibitor cocktail (Thermo Fisher, 78440). Before loading the gel, protein content was determined by Pierce BCA protein assay kit (Thermo Fisher, 23227). Next, the gel was fast-blotted onto a nitrocellulose membrane, using iBlot technology. Membranes were blocked with 3% BSA (Sigma, A7906)-TBST, and incubated with a primary antibody for total ERK (Cell signaling, 4695S) and phospho-ERK (Cell signaling 90101S) and ß-actin (Cell signaling, 3700). After the secondary antibodies (Jackson ImmunoResearch, 111-035-045) were applied on the next day, the membrane was engulfed in HRP solution before it was measured with iBright system.

***In vivo* migration model**

For this experiment, 7-10-week-old Tg(CD2-Il5)5C2Ldt IL-5 transgenic mice (Tg) on the BALB/c background were bred on-site (GZ: BMWFW-66.010-0041WF/II/3b/14). Female and male IL-5 transgenic mice were treated i.p. with SR1001 (25 mg/ kg/ twice a day) or vehicle for 5 times. In vivo chemotaxis of eosinophils was induced by intranasal instillation of 5 µg eotaxin-2/CCL24 (BioTechne, 343-E2). Bronchoalveolar lavage fluid (BALF) and blood was collected 4 hours afterwards, and cell populations were detected by flow cytometry using CD11c-PE-Cy7 (Biolegend, 117330), CD11b-BV421 (Biolegend,

101216), Ly6G-APC (Biolegend 127614) and Siglec-F-PE (Biolegend, 155505) antibodies and further analyzed using the FlowJo 10.8.1 software.

The LabMaster system (TSE Systems, Bad Homburg, Germany) was employed to analyze the effect of SR1001 on the circadian pattern of locomotion, exploration, drinking and feeding in singly housed IL-5Tg mice. Locomotion, exploratory behavior, and water and food intake of the test mice were continuously recorded in the home-like environment of the LabMaster system (TSE Systems, Bad Homburg, Germany) as previously described. For this purpose, transparent LabMaster experimental cages were surrounded by two frames emitting infrared beams to measure vertical exploratory behavior as well as horizontal locomotor activity by counting infrared beam interruptions. In addition, two weight sensors attached to the cage lids were used to assess ingestive behavior as a food container and a drinking bottle were attached to the sensors throughout the experiment. All recording devices were connected to a personal computer, which was used to record and analyze the data using LabMaster software. Food and water intake were recorded in grams of food (g) and milliliters of water (ml) respectively. Before starting the experiment, the animals were habituated to the food containers and the drinking bottles of the LabMaster system as well as to single housing for three days. In the LabMaster system, the mice need to be housed individually to allow accurate activity measurements.

House Dust mite (HDM) model

8-12-week-old female and male BALB/c mice bred on-site, were challenged intranasally with 10 µg of house dust mite allergen (dissolved Acarizax SLIT-tablet) once a week. Following the fourth treatment the mice were treated i.p. with SR1001 (25 mg/ kg/ twice a day) or vehicle every 12 hours, in total for 5 times. Lung function/airway hyperreactivity of these mice was measured by a methacholine challenging test using the FlexiVent platform (Scireq). Additional, bronchoalveolar lavage and blood was collected and cell populations were detected by flow cytometry using CD11c-PE-Cy7 (Biolegend, 117330), CD11b-BV421 (Biolegend, 101216), Ly6G-APC (Biolegend 127614) and Siglec-F-PE (Biolegend, 155505) antibodies and further analyzed using the FlowJo 10.8.1 software [27]. Lungs were either snap-frozen in liquid nitrogen or fixed with formalin for 1 h at room temperature and embedded in paraffin.

On the last day, mice were anaesthetized, ventilated through an endotracheal cannula, and lung function parameters such as airway resistance and lung compliance were measured in response to increasing concentrations of methacholine by using the FlexiVent platform (Scireq).

Periodic-Acid-Schiff (PAS) staining

Mouse lung sections of 5 µm were deparaffinized followed by oxidation in a periodic acid solution (Biognost, BS-OT-1L) for 5 min. Slides were washed in distilled water and placed in Schiff´s reagent (Biognost, BS-OT-1L) for 15 min. Sections were washed for 5 min followed by a counterstain with Myer´s

hematoxylin (Sigma, T865.2). Samples were dehydrated, put in xylene and covered in mounting media. All slides were scanned with the Aperio slide scanner (Leica) analyzed using ImageJ.

Statistical Analyses

Data are shown as mean ± SEM for n observations. Statistical analyses were performed using GraphPad Prism software 6.0 (La Jolla, CA; USA). To identify statistical outliers, Grubbs test was conducted and normal distribution was confirmed by Shapiro-Wilk test. Comparisons between groups were made using either a t-test or Mann-Whitney-U test. One-way or Two-way ANOVA followed by a post hoc test was applied as indicated. Probability values of $p < 0.05$ were considered statistically significant and are indicated as *$p < 0.05$; **$p < 0.01$; ***$p < 0.001$, and ****$p < 0.0001$.

Supplemental Figures

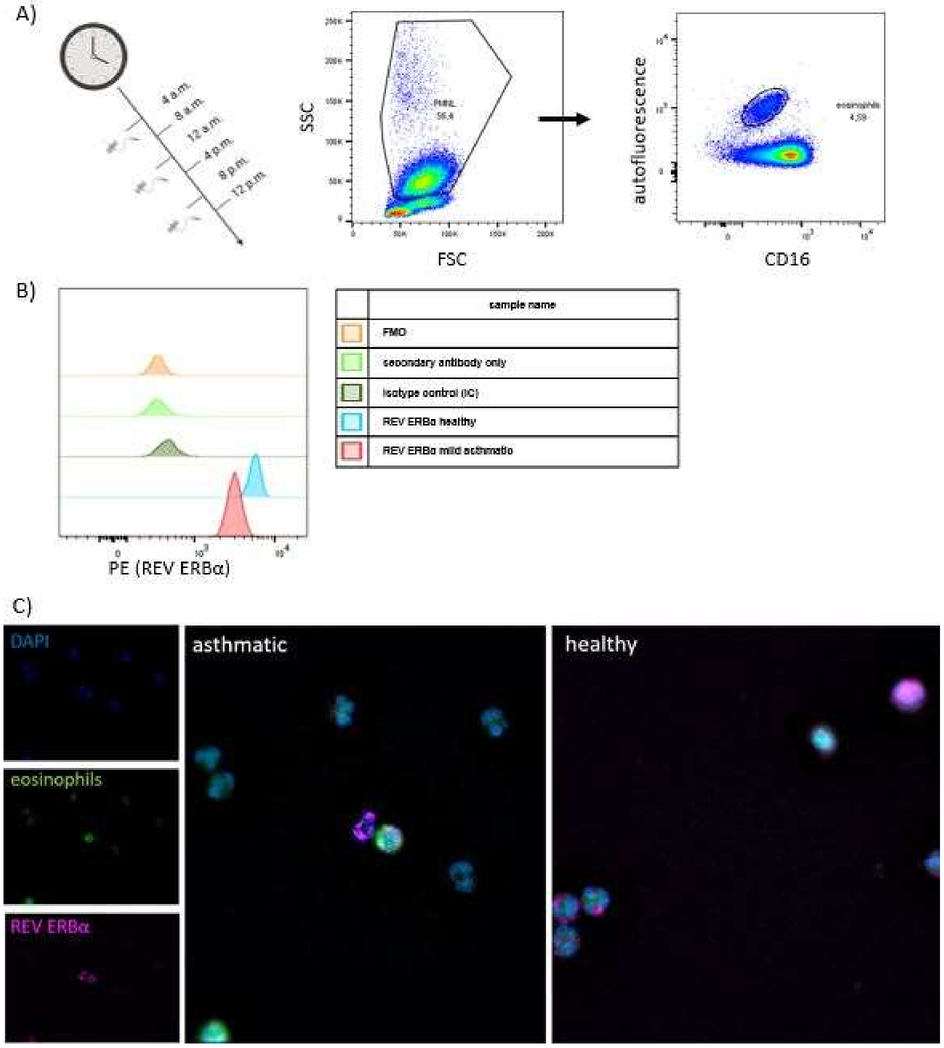

Supplementary Figure 1: Experimental setup and gating. (A) Blood samples were collected three times a day, processed immediately or incubated for 4 hours, resulting in six evaluation time points per day. Eosinophils were identified based on FSC/SSC properties and surface marker staining. (B) Representative FACS histogram of the PE-channel, illustrating FMO, secondary antibody only, isotype control (IC), and REV ERBα antibody staining for a mild asthmatic patient and a healthy donor at 4 a.m. (C) Representative images of blood samples stained for REV ERBα as described above, acquired with an Olympus IX73 fluorescence microscope.

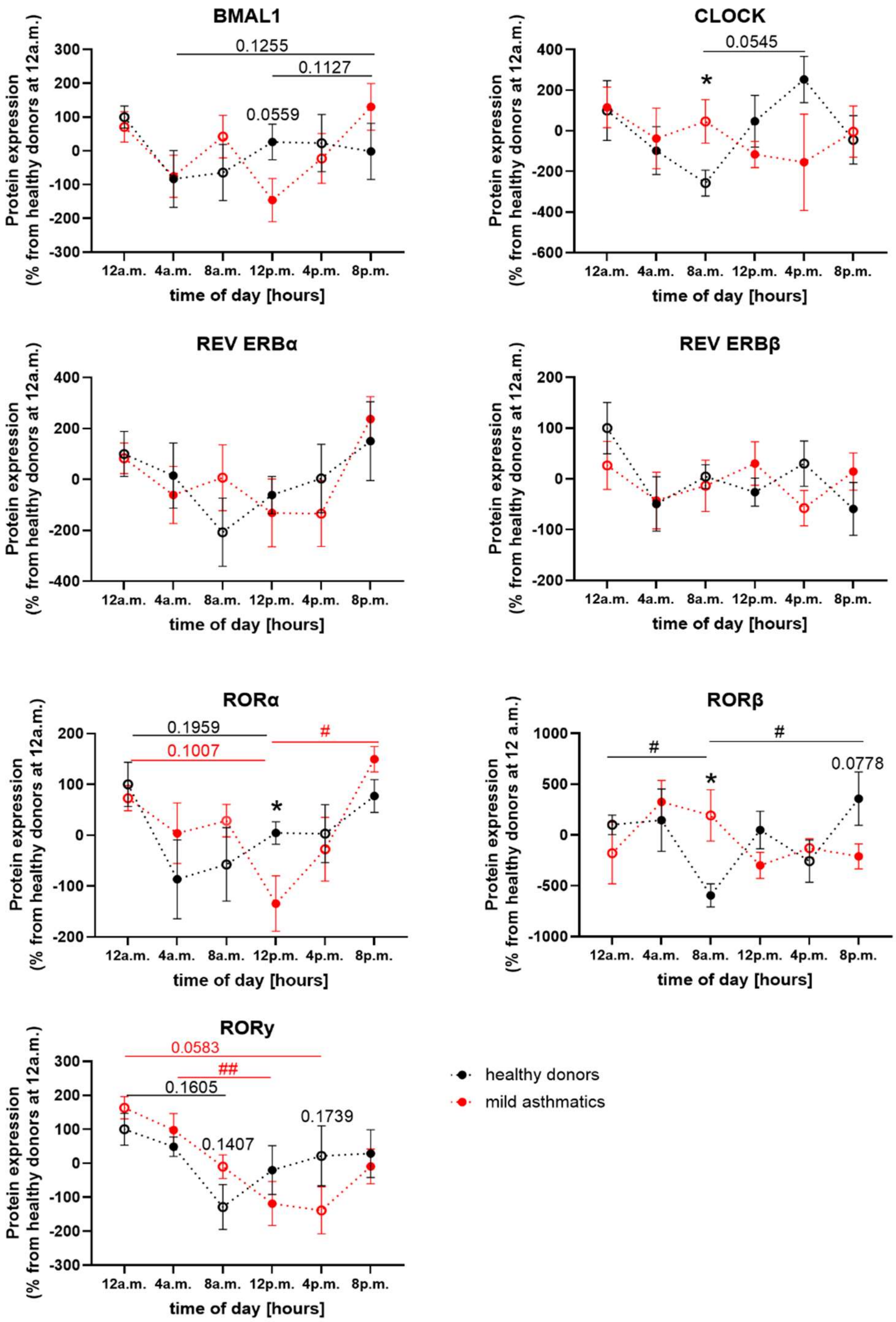

Supplementary Figure 2: Oscillating expression pattern of clock proteins in human peripheral neutrophils. Blood was collected from healthy donors (black, n=9) and mild asthmatics (red, n=8) and BMAL1, CLOCK, REV ERBs and RORs were detected by flow cytometry every 4 hours. Samples analyzed immediately are represented by dots, while samples analyzed after 4 h of incubation are shown as circles. Neutrophils were gated according to FSC/SSC properties and surface marker staining. For statistical analyses, Z-scores were calculated and normalized to the mean of the healthy control group. Paired Two-Way ANOVA, Tukey post hoc test, Comparison within the group is indicated with lines and hashtags (in respective color), while comparison between the two groups is indicated with asterisks. * and # represent $p < 0.05$, ** and ## indicate for $p < 0.01$.

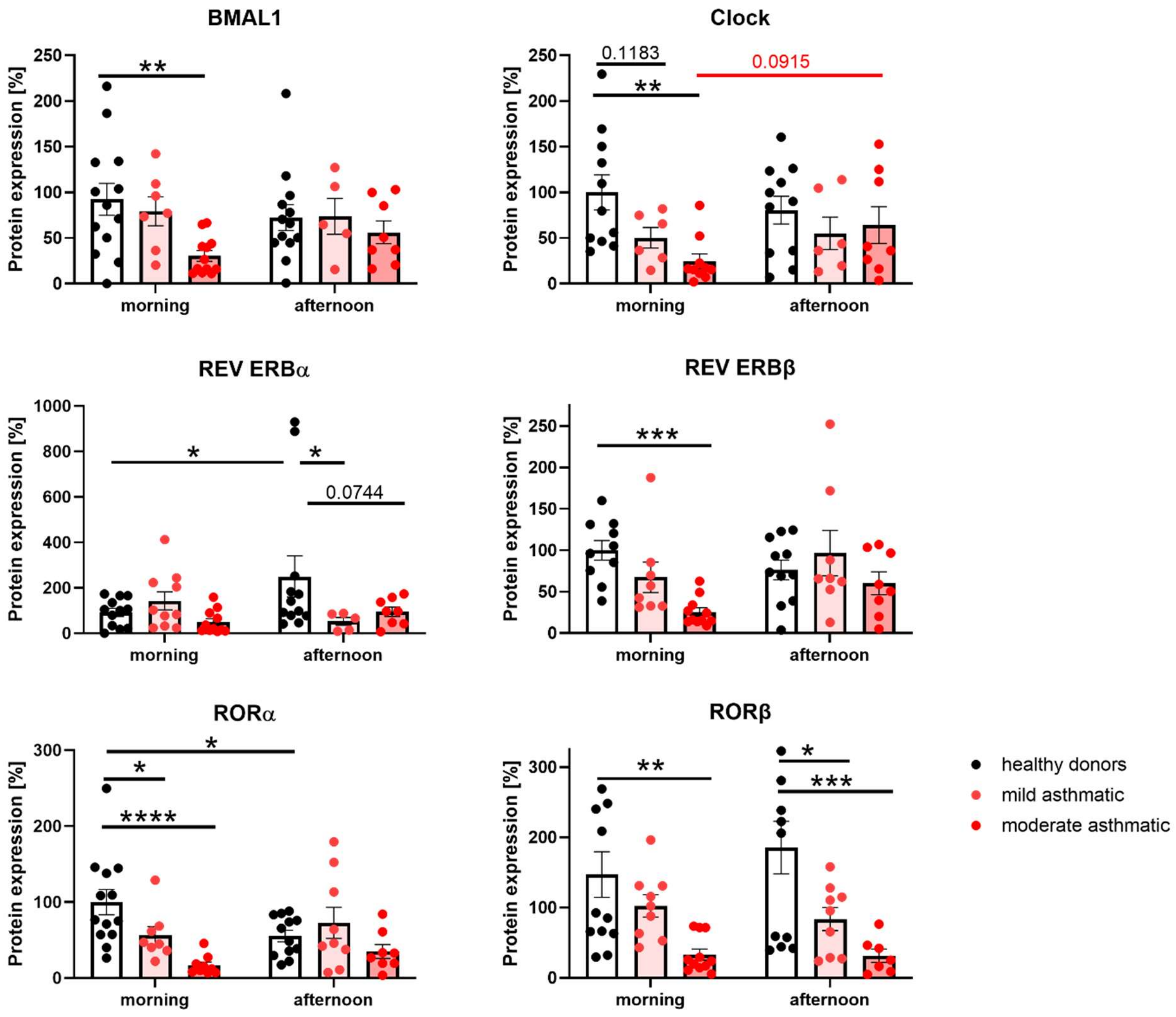

Supplementary Figure 3: Comparison of clock protein levels of healthy donors, mild and moderate asthmatic patients. For the mild asthmatics, protein levels of the 8 a.m. and 12 p.m. time points were averaged and compared to the morning group (9 a.m. – 1 p.m.) of moderate asthmatics, while the 4 p.m. time point of the mild asthmatics was chosen for the comparison with the afternoon group of moderate asthmatics. The data were normalized to the respective control groups. Two-Way ANOVA, * $p < 0.05$, ** $p < 0.01$, *** $p < 0.001$, **** $p < 0.0001$.

A)

moderate asthmatics		Conventional work schedule mean	Conventional work schedule SD	shift worker mean	shift worker SD
participants		12		3	
age		48	19,8	44	10,61
gender	female	8		2	
	male	4		1	
BMI		30	5,21	30	2,20
asthma	uncontrolled	1		1	
	high FeNo	6		1	
spirometry data	FVC predicted	90	19.09	88	4.55
	obstructive	3		2	
blood test	atopic	5		n.a.	
	High blood eosinophils (>0.3*10⁹/L)	8		3	
co-mobilities		5		2	
environmental triggers	mould	2		2	
	pet owner	4		2	
smoking history	(ex-)smoker	5		2	
	pack years	2	3.52	6	9.66
**shift worker**		**0**		**3**	

B)

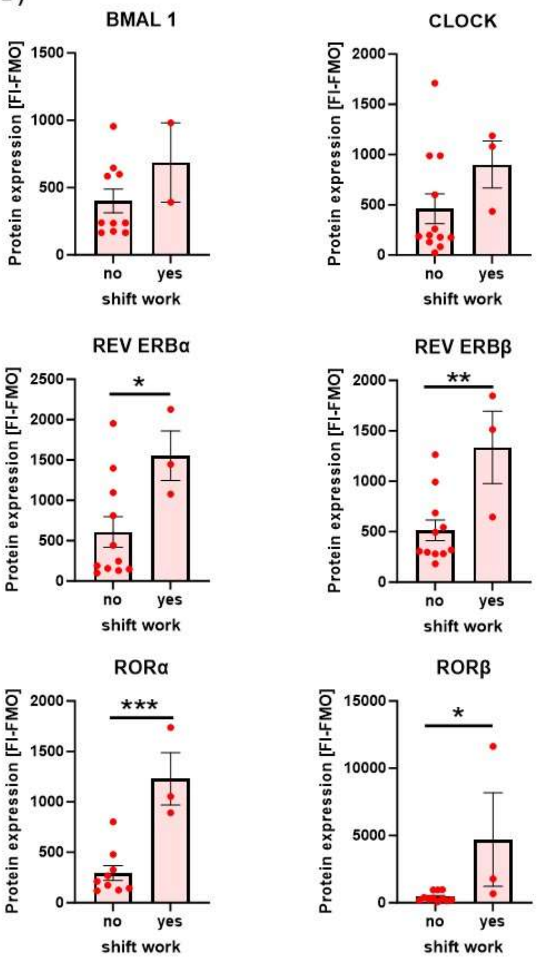

Supplementary Figure 4: Clock protein expression in asthmatic patients on shift work. (A) Demographic table of moderate asthmatics from the morning group with conventional work schedule (n=12) or working shifts (n=4). (B) Significant higher protein expression of the receptor families REV ERBs and RORs was observed in shift working patients compared to those having a conventional working schedule. T-test, * $p < 0.05$, ** $p < 0.01$, *** $p < 0.001$.

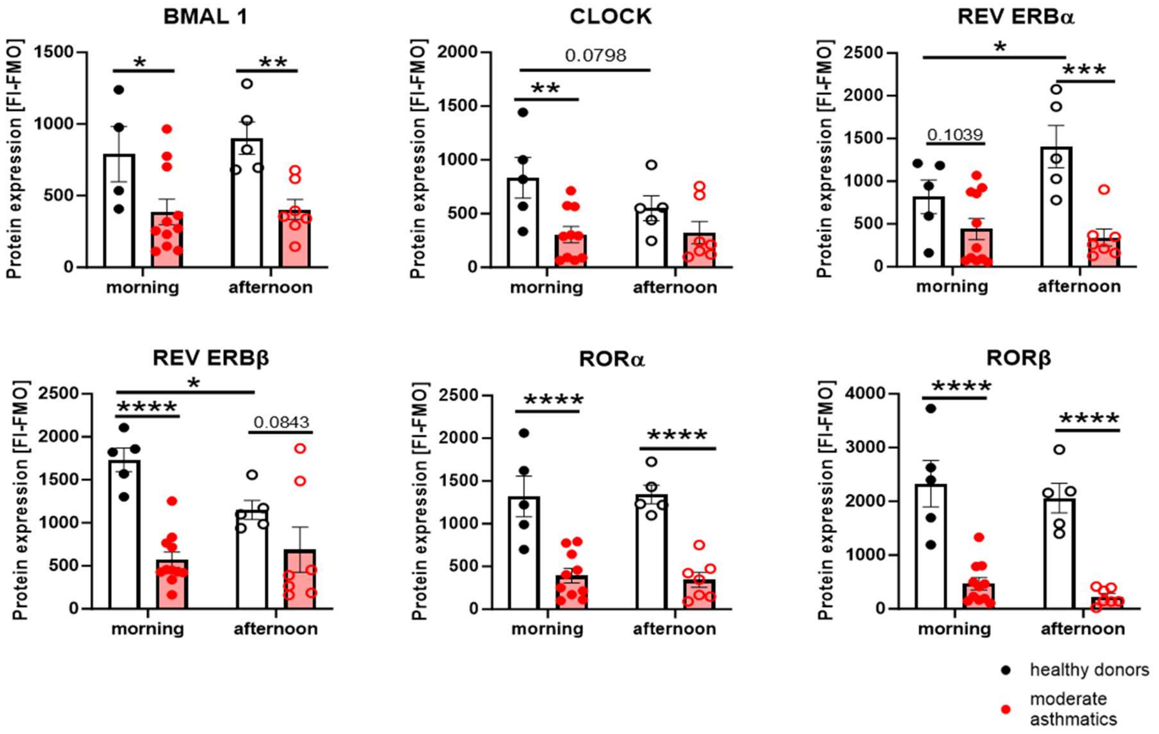

Supplementary Figure 5: Clock protein expression is damped in peripheral neutrophils of moderate asthmatic patients. All participants were allocated to the morning (filled dots) or afternoon (empty circles) group depending on the time of evaluation. Significant lower circadian protein expression was observed in neutrophils derived from moderate asthmatics (red, n=19) compared to healthy donors (black, n=10) in both groups. Shapiro-Wilk test, Two-Way ANOVA, * $p < 0.05$, ** $p < 0.01$, *** $p < 0.001$, **** $p < 0.0001$.

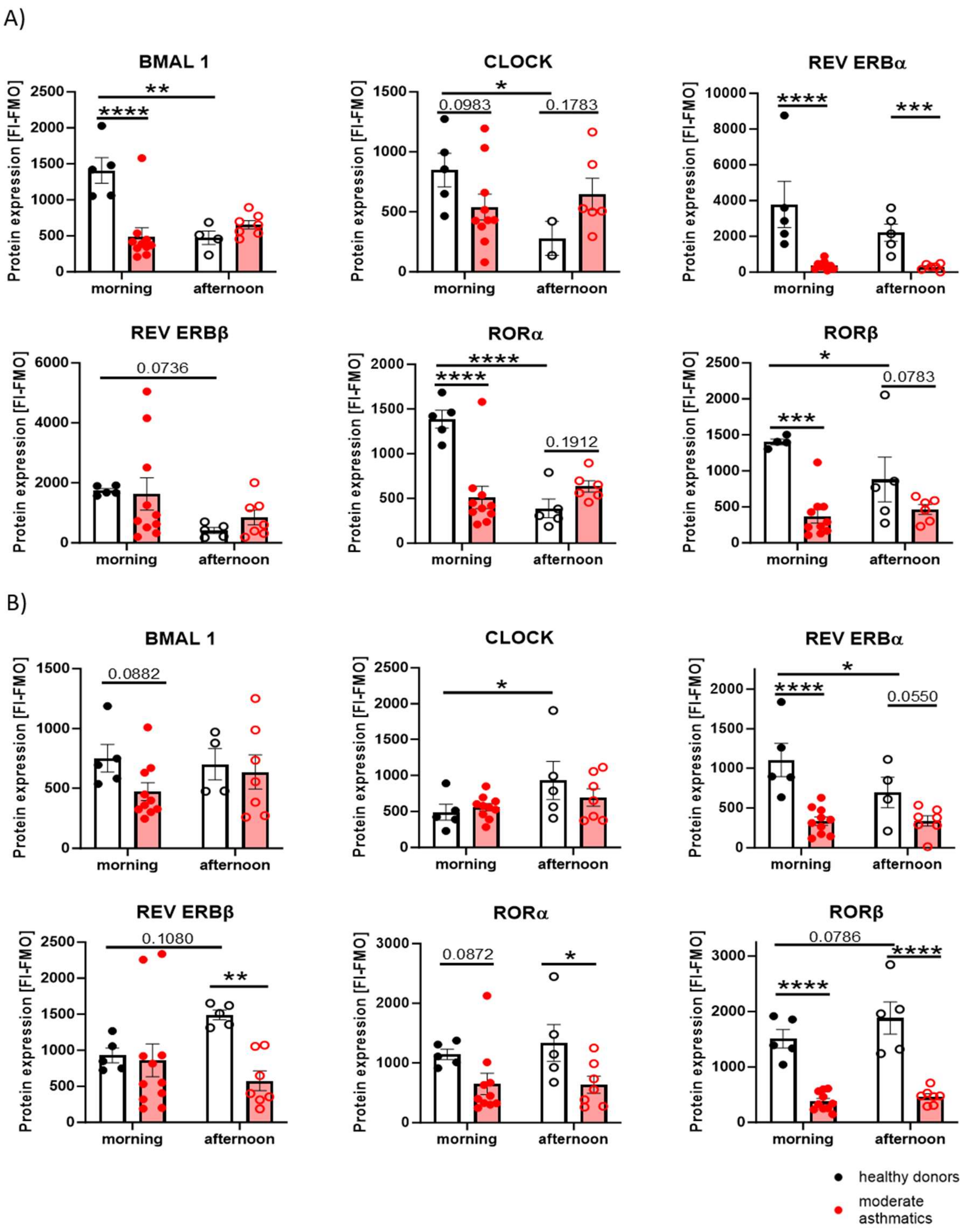

Supplementary Figure 6: Clock protein expression is damped in peripheral monocyte subsets from patients with moderate asthma. All participants were allocated to the morning (filled dots) or afternoon (empty circles) group depending on the time of evaluation. Significant lower circadian protein expression was observed in (A) CD14+CD16- monocytes and (B) CD14+CD16+ monocytes derived from moderate asthmatics (red, n=19) compared to healthy donors (black, n=10) in both groups. Shapiro-Wilk test, Two-Way ANOVA, * $p < 0.05$, ** $p < 0.01$, *** $p < 0.001$, **** $p < 0{,}0001$.

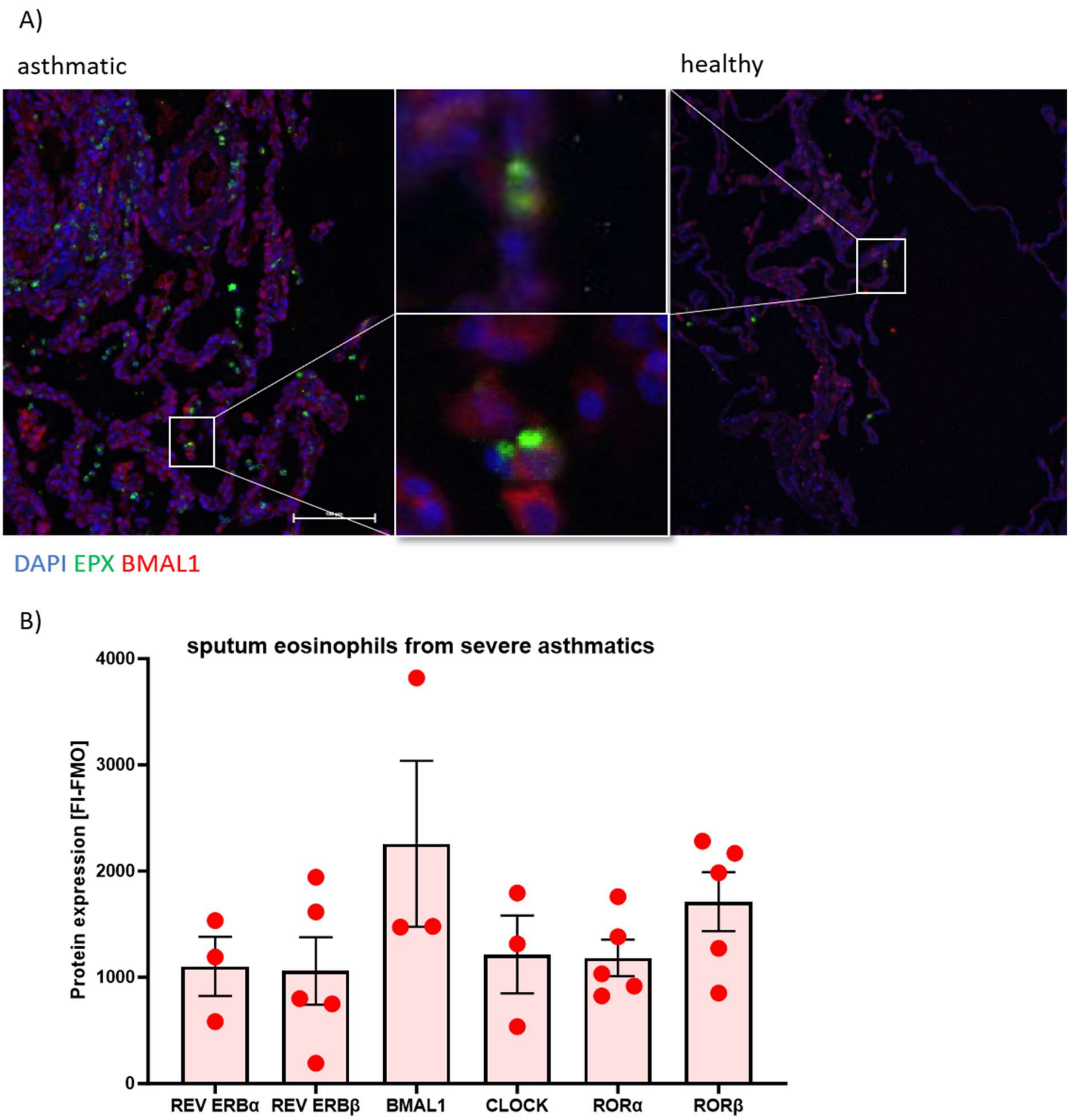

Supplementary Figure 7: Clock proteins are expressed in tissue and sputum eosinophils of asthmatics. (A) Biopsies from asthmatics and healthy controls were stained with anti-EPX for eosinophils and anti-BMAL1. Representative images are shown (scale bar 100 μm). EXP is shown in green and BMAL1 is labeled in red (n≥3). (B) Cells were isolated with DTT from fresh spontaneous sputum samples from severe asthmatic patients. Samples were stained for surface markers and intracellular clock proteins by flow cytometry (n=4).

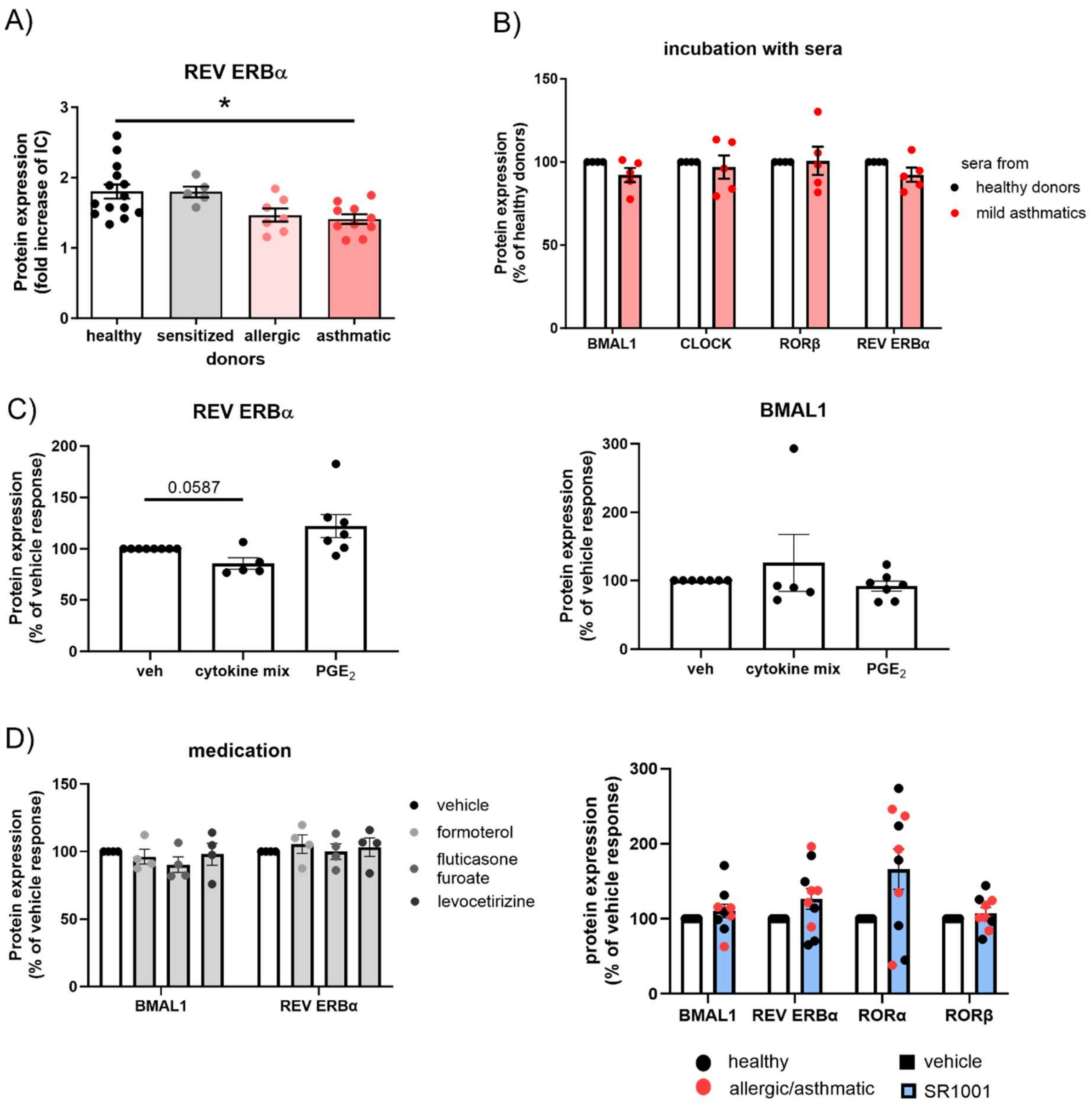

Supplementary Figure 8: Bidirectional interplay between the circadian clock and neutrophil responsiveness. (A) REV ERB*α* expression is decreased only in neutrophils derived from blood donors with mild asthma compared to healthy controls (n≥5). (B) Stimulation of neutrophils from healthy donors with sera from mild asthmatics had no effect on clock protein expression (n≥5). (C) Stimulation of PMNL from healthy donors with a pro-inflammatory cytokine mix partly reduced REV ERB*α* (n≥5). (D) Incubation with asthma/ allergy medication does not alter the peripheral clock of eosinophils (n=4). (E) SR1001-induced increase in the expression of clock proteins did not reach statistical significance (n=10). unpaired t-test or Mann-Whitney test, One-Way or two-Way ANOVA, * p < 0.05.

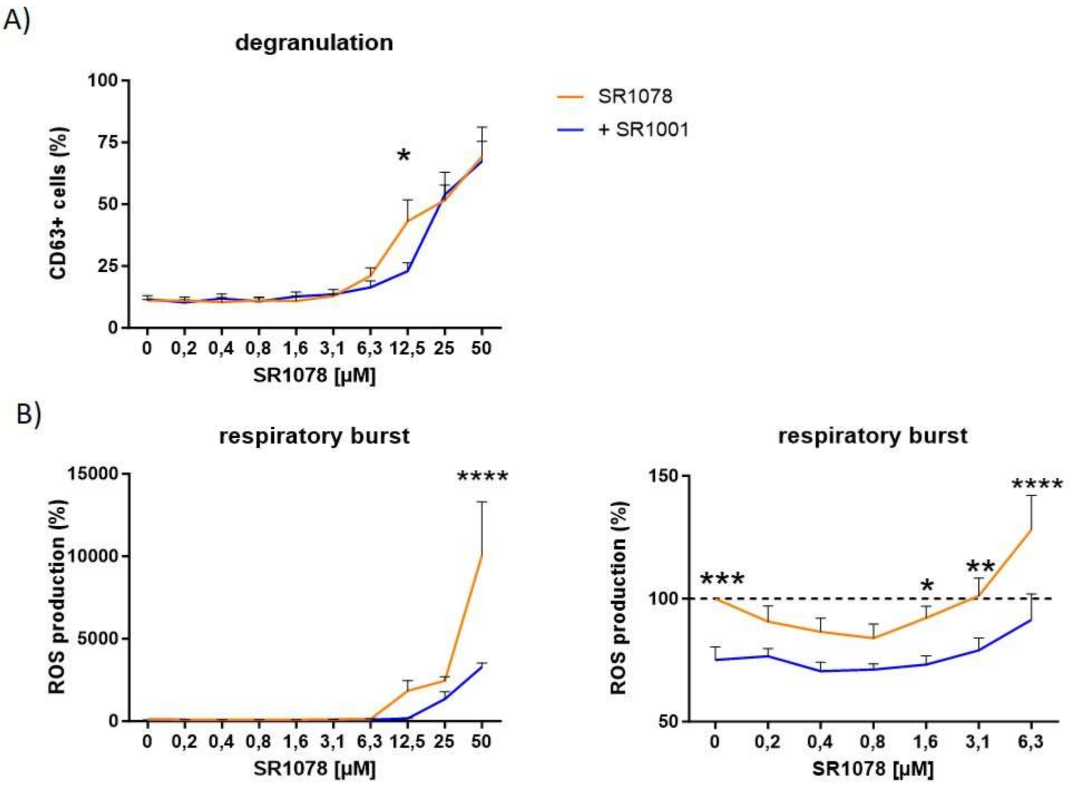

Supplementary Figure 9: The ROR agonist SR1078 contracts the effects of the inverse ROR agonist SR1001 have opposing effects on eosinophil effector functions. Purified eosinophils were pretreated with 10 µM SR1001 for 3 hours. (A) Cells were mixed with 5 µg/ml cytochalasin B and stained with FITC-anti-CD63 to measure degranulation using flow cytometry (n=8). (B) To explore the influence on ROS production a dihydrorhodamine-123-based respiratory burst assay was performed (n=10). Repeated Two-Way ANOVA, Šídák's post hoc test. * $p < 0.05$, ** $p < 0.01$, *** $p < 0.001$, **** $p < 0.0001$.

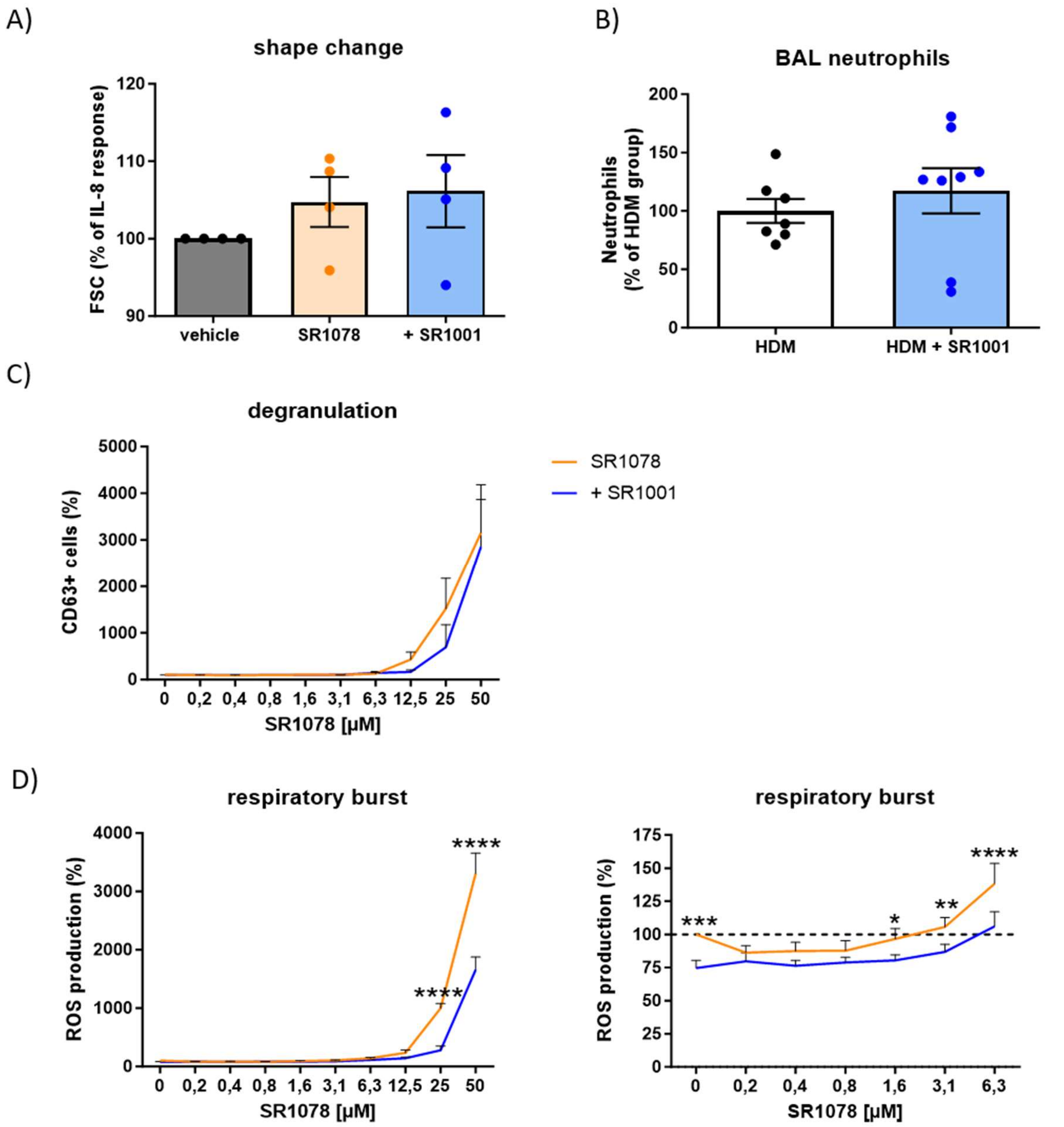

Supplementary Figure 10: ROR signaling triggers ROS production in neutrophils. (A, C and D) PMNL were pre-treated with 10 µM SR1001 and/or treated with 10 µM SR1078. (A) After the treatment, a shape change assay towards IL-8 was performed (n=5). (B) BALB/c mice were treated i.n. with HDM extract (10 µg) or vehicle once a week for four weeks. Starting from day 22, after a 3-day acclimatization period in the LabMaster cages, mice were treated 5 times with 25 mg/kg SR1001 i.p. After the last SR1001 injection on day 24, BAL was collected (n≥7). (C) Cells were mixed with 5 µg/ml cytochalasin B and stained with FITC-anti-CD63 to measure degranulation using flow cytometry (n=8). (D) To explore the influence on ROS production a dihydrorhodamine-123-based respiratory burst assay was performed (n = 10). One Way ANOVA or repeated Two-Way ANOVA, Šídák's post hoc test. * $p < 0.05$, ** $p < 0.01$, *** $p < 0.001$ **** $p < 0.0001$.

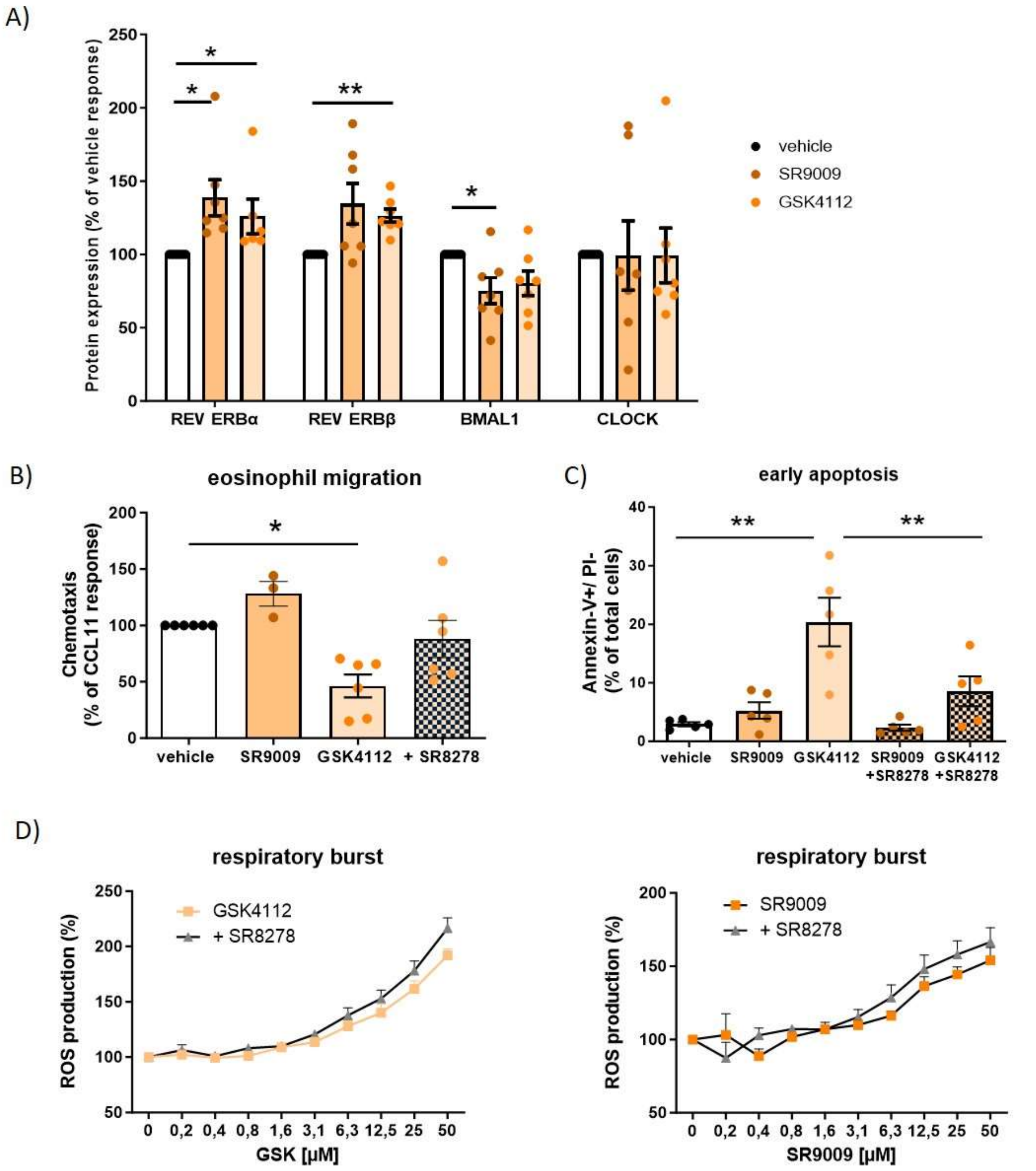

Supplementary Figure 11: GSK4112 and SR9009-induced effects on eosinophil effector function are partially independent of REV ERB*α*. (A) PMNL were treated with 10 μM of the respective REV ERB agonist for 3 hours and expression levels of the clock proteins REV ERB*α*, REV ERBβ and BMAL1 were detected by intracellular flow cytometric staining, eosinophils were gated by their FSC/SSC properties and autofluorescence (n≥7). PMNL or purified eosinophils were pretreated with 10 μM SR8278, treated with 10 μM SR9009 or GSK4112 for 3 hours. (B) Eosinophil migration assay was performed towards 3 nM eotaxin-1/CCL11 in a microBoyden chemotaxis chamber (n>3). (C) Eosinophils were stained with APC-Annexin-V and Propidium iodide. Samples were immediately analyzed by flow cytometry (acquisition set for 60s at medium flow rate, n=5). (D) ROS production was quantified by flow cytometry using a dihydrorhodamine-123-based assay (n=5). One-Way ANOVA or two-Way ANOVA, *p < 0.05, ** p < 0.01.

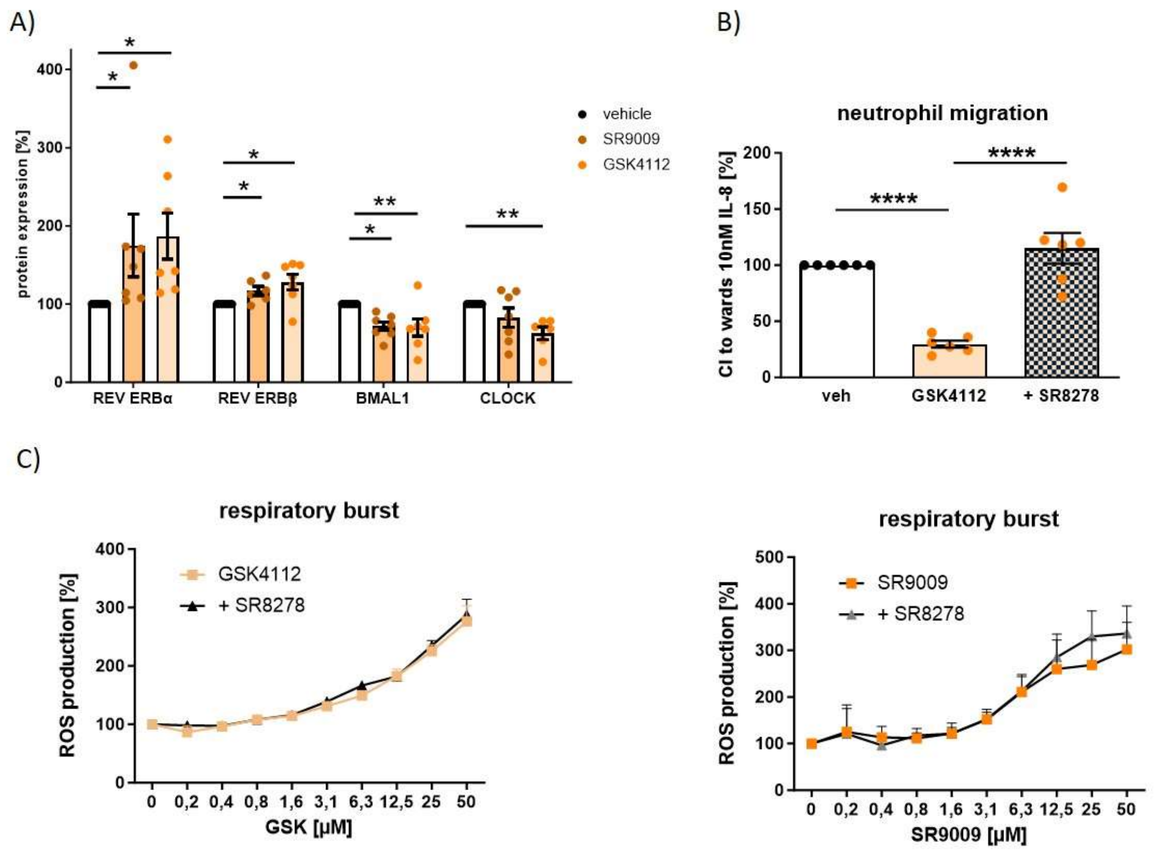

Supplementary Figure 12: GSK4112 and SR9009-induced effects on neutrophil function are partially independent of REV ERBα. (A) PMNL were treated with 10 μM of the respective REV ERB agonist for 3 hours and expression levels of the clock proteins REV ERBα, REV ERBβ and BMAL1 were detected by intracellular flow cytometric staining (n≥7). PMNL were pretreated with 10 μM SR8278 and treated with 10 μM SR9009 or GSK4112 for 3 hours. (B) Neutrophil migration assay was performed towards 10 nM IL-8 in a microBoyden chemotaxis chamber (n=6). (D) ROS production was quantified by flow cytometry using a dihydrorhodamine-123-based assay (n=5). One-Way ANOVA or two-Way ANOVA, * $p < 0.05$, ** $p < 0.01$, **** $p < 0.0001$.
